# Gradient boosted regression as a tool to reveal key drivers of temporal dynamics in a synthetic yeast community

**DOI:** 10.1093/femsec/fiae080

**Published:** 2024-05-22

**Authors:** Cleo Gertrud Conacher, Bruce William Watson, Florian Franz Bauer

**Affiliations:** Department of Viticulture and Oenology, South African Grape and Wine Research Institute, Private Bag X1, Stellenbosch University, Stellenbosch 7600, South Africa; Centre for Artificial Intelligence Research (CAIR), School for Data-Science & Computational Thinking, Stellenbosch University, Stellenbosch 7600, South Africa; Centre for Artificial Intelligence Research (CAIR), School for Data-Science & Computational Thinking, Stellenbosch University, Stellenbosch 7600, South Africa; Department of Viticulture and Oenology, South African Grape and Wine Research Institute, Private Bag X1, Stellenbosch University, Stellenbosch 7600, South Africa

**Keywords:** machine learning, synthetic ecology, yeast ecosystems

## Abstract

Microbial communities are vital to our lives, yet their ecological functioning and dynamics remain poorly understood. This understanding is crucial for assessing threats to these systems and leveraging their biotechnological applications. Given that temporal dynamics are linked to community functioning, this study investigated the drivers of community succession in the wine yeast community. We experimentally generated population dynamics data and used it to create an interpretable model with a gradient boosted regression tree approach. The model was trained on temporal data of viable species populations in various combinations, including pairs, triplets, and quadruplets, and was evaluated for predictive accuracy and input feature importance. Key findings revealed that the inoculation dosage of non-*Saccharomyces* species significantly influences their performance in mixed cultures, while *Saccharomyces cerevisiae* consistently dominates regardless of initial abundance. Additionally, we observed multispecies interactions where the dynamics of *Wickerhamomyces anomalus* were influenced by *Torulaspora delbrueckii* in pairwise cultures, but this interaction was altered by the inclusion of *S. cerevisiae*. This study provides insights into yeast community succession and offers valuable machine learning-based analysis techniques applicable to other microbial communities, opening new avenues for harnessing microbial communities.

## Teaser

An interpretable machine learning model revealed which inoculation parameters are most important to consider for understanding and manipulating community succession in a synthetic wine yeast community.

## Introduction

Microbial communities are ubiquitous in nature and foundational to all living systems. Understanding the underlying ecological interactions and cellular and molecular functioning of microbial communities is one of the major remaining research frontiers in microbiology (Altermann and Hickey 2020, Sun and Sanchez 2023). The role of time has been highlighted as a critical lens through which to unravel fundamental principles of ecological systems (Ryo et al. 2019). Given that microbial communities are dynamic entities, investigating these communities through a temporal framework is essential for understanding their succession, adaptation, and resilience. Furthermore, in the rapidly developing field of leveraging predictive modelling for biological applications, integrating principles of temporal dynamics into the development of predictive models for microbial communities is timely.

There is a paucity of research into eukaryotic microbial communities, and as such, comparatively very little is known currently about how eukaryotes interact and function within microbial communities when compared to prokaryotes (Conacher et al. 2019). A eukaryotic microbial community of industrial significance is the community of yeasts involved in the natural fermentation of grape must to wine, referred to as the wine yeast community. The diversity and composition of the yeast community involved in wine fermentations impact the final properties of wine; however, the causative mechanisms involved are still largely viewed as a black box, which has driven research into understanding how the wine yeast community functions. Besides the commercial relevance, from a fundamental research perspective, the wine yeast community has several properties that make it an ideal model for studying eukaryotic microbial community functioning (Conacher et al. 2021). Fermenting grape must is a harsh environment for yeasts, with stressors of a physical and chemical nature (e.g. low pH, high osmolarity, limited nitrogen sources, ethanol) as well as stressors of a biotic nature (several competing populations of yeast species) (Bauer and Pretorius 2000, Conacher et al. 2019). Since wine is an exclusively anthropogenically linked product, humans have influenced these yeasts’ evolution (i.e. domestication) as we have selected for adaptations that are advantageous for successful fermentation. Notably, wine yeasts are phylogenetically separate from environmental isolates, as well as beer- and bread-associated yeast isolates; we have thus created an evolutionary isolated niche (Chambers et al. 2015, Gibbons and Rinker 2015). This is additionally evidenced phenotypically since wine yeasts are inherently better adapted to fermentation-relevant stressors and excel at functions related to fermentation (Guillamón and Barrio 2017). Further, there is global uniformity of wine yeast community members, implying relatively consistent biotic selection pressures for community member coevolution (Belda et al. 2021). Therefore, the wine yeast community provides a consistent ecological framework from which to study eukaryotic microbial community functioning and dynamics (Conacher et al. 2021, Ruiz et al. 2023).

Temporal patterns are central to the wine fermentation process. During natural or spontaneous wine fermentation, the yeast community is introduced to the grape must at the beginning of the process, referred to here as ‘time zero’. Time zero of the fermentation is therefore arguably the most determinant biological phase of natural wine fermentations. From an industrial perspective, this is also the point at which interventions are most practical to make to ensure successful completion of the fermentation process. There is a large body of work regarding description of yeast community succession that follows this initial inoculation, where temporal abundance changes of different yeast species populations have been mapped through time (Comitini et al. 2011, Ciani and Comitini 2015, Bokulich et al. 2016, Bagheri et al. 2017, Bagheri et al. 2018, 2020, Alonso-del-Real et al. 2019). The consensus is that the community undergoes a decrease in diversity, until one dominant species remains: *Saccharomyces cerevisiae* (Conacher et al. 2021, Ruiz et al. 2023). This is especially interesting from an ecological standpoint since the abundance of *S. cerevisiae* in the natural community at the start of fermentation is very low, yet it consistently outcompetes more abundant yeast species populations. There is a good consensus regarding the fact that temporal succession occurs during wine fermentation, and that the diversity and composition of the wine yeast community at different stages of fermentation cause chemical changes to the final wine product; however, a predictive understanding of this process has not been achieved.

Further, since time zero is so pivotal to this process, there is an opportunity to investigate how the environment of these yeasts prior to time zero may impact on this succession. This concept is loosely captured in the definitions of ‘ecological memory’ and ‘priming effects’ (Ryo et al. 2019). Within the context of this paper, we are able to manipulate the environment of members of the wine yeast community prior to their introduction to an experimental community. Thanks to a wealth of fundamental physiological research on the model organism *S. cerevisiae*, we know that the environment that a yeast is cultured in, i.e. growth media, will impact on its metabolism, and similarly, the metabolic state of yeasts is also impacted in each phase of growth in a batch experiment (Bely et al. 2005, Kolkman et al. 2006, Lackner et al. 2012, Hall et al. 2014, Thomas 2015, Kragh et al. 2018, Keil et al. 2019). By manipulating the growth medium and growth phase of yeast communities prior to their inoculation into fermentation, it can roughly shed light on to what extent the history of a particular yeast needs to be considered in the prediction and control of succession in the wine yeast community.

The diversity of the natural wine yeast community is such that disentangling the causative mechanisms behind how each member, as well as ecological interactions between members, ultimately results in a particular fermentation outcome is an enormously complex theoretical and experimental challenge. In synthetic ecology, thoughtfully designed subsets of natural ecosystems are studied to reduce the technical complexity to a level where accurate deductions can be made regarding ecosystem establishment and functioning (Ben Said and Or 2017, Ruiz et al. 2023). In particular, bottom-up approaches of building simplified wine yeast communities are common to investigate yeast inter-species interactions. Interactions are defined here as changes to a yeast’s physiology that occur exclusively when exposed to a different yeast species population (i.e. are different from what occurs during mono-species culture). Within this context, most commonly, transcriptomic and proteomic approaches combined with population dynamics have been taken to investigate pairwise inter-species interactions (Curiel et al. 2017, Tronchoni et al. 2017, Peng et al. 2019, Shekhawat et al. 2019, Bordet et al. 2020, Tondini et al. 2020, Mencher et al. 2021), and these inter-species interactions have a clear impact on temporal dynamics of the synthetic yeast communities. However, recent evidence suggests that additive pairwise inter-species interactions alone cannot predict how more complex yeast communities, which more accurately resemble the natural diversity, will perform (Conacher et al. 2022, Chang et al. 2023). Further, there is currently a research chasm in the study of synthetic wine yeast communities, where there has been focus on either two species or much larger assemblages (n species > 7), but very limited work on synthetic communities between these sizes and even less on evaluating impacts of stepwise increases in diversity. This is problematic because we know that species-specific interactions occur; therefore, teasing apart the trajectory of complex communities based on their initial composition is not possible at this point since we do not know enough about how assembly impacts future population dynamics. Therefore, if we are to gain a predictive understanding of community dynamics, synthetic communities of more than two species need to be studied further. This is, however, easier said than done.

There are significant challenges that accompany investigation of complex microbial communities and their associated interactions (Widder et al. 2016). Firstly, exhaustively investigating interactions within a microbial community in different environmental settings generates an experimental space that is usually logistically unfeasible to execute. Secondly, in cases where researchers do indeed attempt complex microbial community experiments, the data generated are rich and challenging to derive meaning from in an objective way, especially considering that these data largely do not conform to the assumptions for traditional statistical tests.

Machine learning (ML) has emerged as a promising tool in microbial community research (Rubbens and Props 2021). Studies applying ML algorithms to experimental microbial community data are limited, with most examples either using simulated microbial community data and/or focusing on pairwise interactions alone. The generation of multivariate time series models from tabular datasets, such as those commonly generated in microbial ecology studies, is an active research challenge in the ML field, with most benchmarking having been performed for univariate time series (Padhi et al. 2021). Nevertheless, for multivariate dynamic tabular data, tree-based methods are the current recommendation in terms of performance, with neural network architecture performance lagging behind (Borisov et al. 2022). Tree-based algorithms, which involve various conformations and combinations of decision trees, have been shown to perform well on available microbial community datasets. These studies have ranged from predicting strength and direction in pairwise interactions of bacteria (DiMucci et al. 2018), prediction of hydrogen sulphide production (Dutta et al. 2022) and dissolved organic carbon levels (Thompson et al. 2019) based on microbial community structure as an input, prediction of soil microbial community structure from environmental parameters (Peng et al. 2022), and ranking of phenotypes important to sugar consumption in wine yeast communities (Ruiz et al. 2023). It is therefore clear that these algorithms may aid in the quest to predicting temporal dynamics in microbial communities. In this study, we have opted for a Gradient Boosted Regression (GBR) tree-based model. In GBR models, during the training process, a given loss function is optimized by sequentially adding decision trees, in a way that minimizes the residual errors from previous iterations, ultimately generating an ensemble of decision trees that, if properly tuned, accurately predicts target values (Friedman 2002).

Here, we explored which factors impact temporal dynamics and succession of a synthetic wine yeast community, using concepts of past (prior to time zero), present (at time zero), and future (after time zero) by applying an explainable ML algorithm. A combination of near-exhaustive multi-species experimental cultures of a 4-species synthetic yeast community with the generation of a tree-based GBR model was applied. Time series of community composition and absolute viable cell numbers were generated in species pairs, triplets, and quadruplets using four different yeast population ‘history’ settings prior to growth in the community.

We sought to make use of the GBR model to provide insights into three biologically relevant elements that may impact community succession: (i) whether different pre-culture conditions would impact growth of community members in a competitive environment, showing whether the initial physiological state of yeast cells impact on competitive growth phenotypes, (ii) the importance of inoculation dosage as opposed to inoculation alone in impacting community member performance, indicating the impact of initial population size in community dynamics; within this context, we also evaluated how inoculation dosage of other species impact each community member, and finally, (iii) how congruent important model features are between pairwise (*n* = 2) combinations versus more complex (*n* > 2) combinations, to show emerging properties of complex systems such as higher-order multi-species interactions. These factors are readily implementable in the context of understanding and manipulating temporal dynamics of microbial communities by adjusting procedures related to the point of inoculation.

## Materials and methods

### Yeast strains

Four yeast species representatives of wine-related origin were used to construct model yeast communities of various complexities and species combinations as in Table 1. Three of these species were fluorescently labelled, each with a different fluorescent label, while the fourth species was not labelled. The strains representing the four species were: *S. cerevisiae* VIN13 (Anchor Yeast, Cape Town, South Africa; biogeographical origin: South Africa) labelled with TagRFP657; *Lachancea thermotolerans* IWBT Y1240 [CBS: 16 374, biogeographical origin: South Africa] labelled with mTagBFP2; *Torulaspora delbrueckii* LO544 [CRBO: LO544, biogeographical origin: France] labelled with eGFP; and unlabelled *Wickerhamomyces anomalus* IWBT Y934 (CBS: 16 372, biogeographical origin: South Africa) (Conacher et al. 2020). All yeast strains were stored as glycerol stocks (25% w/v glycerol) at −80°C.

**Table 1.** Multispecies culture configurations that were conducted to generate the dataset.

Combination of yeast species representatives	Pre-culture in SGM and harvested at exponential phase	Pre-culture in SGM and harvested at stationary phase	Pre-culture in YPD and harvested at exponential phase	Pre-culture in YPD and harvested at stationary phase
*Saccharomyces cerevisiae* + *L. thermotolerans*	X (*n = 2*)	X (*n = 4*)	X (*n =4*)	X (*n = 4*)
*Saccharomyces cerevisiae* + *T. delbrueckii*	X (*n = 4*)	X (*n = 4*)	X (*n = 4*)	X (*n = 4*)
*Saccharomyces cerevisiae* + *W. anomalus*	X (*n = 4*)	X (*n = 4*)	X (*n = 4*)	X (*n = 4*)
*Lachancea thermotolerans* + *T. delbrueckii*	X (*n = 4*)	X (*n = 4*)	X (*n = 4*)	X (*n = 4*)
*Lachancea thermotolerans* + *W. anomalus*	X (*n = 2*)		X (*n = 4*)	X (*n = 4*)
*Torulaspora delbrueckii* + *W. anomalus*	X (*n = 2*)		X (*n = 4*)	X (*n = 4*)
*Saccharomyces cerevisiae* + *L. thermotolerans* + *T. delbrueckii*	X (*n = 5*)	X (*n = 3*)	X (*n = 5*)	X (*n = 3*)
*Saccharomyces cerevisiae* + *L. thermotolerans* + *W. anomalus*		X (*n = 4*)	X (*n = 4*)	X (*n = 4*)
*Saccharomyces cerevisiae* + *T. delbrueckii* + *W. anomalus*	X (*n = 4*)	X (*n = 4*)	X (*n = 4*)	X (*n = 4*)
*Lachancea thermotolerans* + *T. delbrueckii* + *W. anomalus*			X (*n = 4*)	X (*n = 4*)
*Saccharomyces cerevisiae* + *L. thermotolerans* + *T. delbrueckii* + *W. anomalus*		X (*n = 3*)	X (*n= 9*)	X (*n = 7*)

### Growth media

Prior to inoculation, glycerol stocks were streaked out onto Wallerstein Laboratory (WL) nutrient agar (Sigma–Aldrich, Johannesburg, South Africa) and incubated at 30°C for three days. The Synthetic Grape Must (SGM) used here contained: 100 g l^−1^ glucose, 100 g l^−1^ fructose, 200 mg l^−1^ assimilable nitrogen, trace elements and vitamins as described by Henschke and Jiranek (1993) and 10 mg l^−1^ ergosterol.

### Different pre-culture comparisons

All experiments were completed independently of each other, with a biological repeat defined as a culture originating from a separate single colony.

Four of the most commonly reported pre-culture conditions in wine synthetic community experiments were tested, namely, pre-culture in Yeast Peptone Dextrose (YPD) and harvested at either exponential or stationary phase, or pre-culture in SGM and harvested at either exponential or stationary phase. Single colonies of each yeast strain were inoculated into 5 ml of YPD broth (Sigma–Aldrich, Johannesburg, South Africa) in a test tube and incubated on a test tube rotator at 30°C for 18 h. The culture was transferred to 50 ml YPD or SGM, at a concentration of 1 × 10^6^ cells ml^−1^, in a 250 ml Erlenmeyer flask with a cotton plug and foil covering. The flask was incubated at 30°C with agitation (150 RPM) until either exponential (10 h) or stationary phase (24 h) had been reached, after which the culture was harvested and inoculated into multi-species culture. The time at which the pre-cultures were deemed to be in exponential or stationary phase was based on previous monoculture growth curves (Supplementary Fig. S1).

### Community culture inoculation and growth procedure

Pre-cultures were centrifuged at 5000 × *g* for 5 min at room temperature and re-suspended in Phosphate Buffered Saline (PBS), pH 7.2, at a volume of 10× less than the initial culture volume before being enumerated and inoculated into one of 38 multispecies culture options (Table 1). While this is not a full-factorial experimental design, it is still a comprehensive set of experiments that were sufficient for the purpose of this study. All multi-species cultures were conducted as previously described (Conacher et al. 2020) in SGM, at a final volume of 6 ml in a sterile 6-well tissue culture plate, which was sealed with parafilm and incubated at 30°C, with agitation (150 RPM). Each representative species was inoculated in varied cell ratios, for a final total concentration of approximately 3 × 10^6^ cells/ml^−1^, measured by volumetric cell counts using the CytoFLEX (Beckman Coulter) flow cytometer. Monocultures of each species were grown in parallel and were inoculated at an initial concentration of 3 × 10^6^ cells/ml^−1^ (Supplementary Fig. S2).

### Monitoring community population dynamics

Community population sizes, represented by viable cell numbers, were determined by quantitative flow cytometry as previously described (Conacher et al. 2022). Briefly, fluorescently labelled yeast species’ cells are distinguished by fluorescence flow cytometry and volumetric cell concentrations are determined. Samples of 50 µl were taken at time points 0, 6, 12, 24, 48, and 72 h to quantitate viable cell numbers of each species within the community and in monocultures. Samples were diluted in PBS supplemented with EDTA [0.1 M] and propidium iodide [1 µM], prior to flow cytometry analysis.

### Model generation

#### Full community dataset as input

##### Data pre-processing

The first step in generating the model is to convert the dataset into a format that is computationally readable. Here, the dataset was represented as a dynamic tabular dataframe (Supplementary Dataset File). This dataframe was generated by performing data pre-processing. The categorical variables of pre-culture conditions were transformed using one-hot encoding, a data pre-processing method that converts a categorical variable into a binary numerical format that is readable as an input for a model, as follows: two mutually exclusive one-hots representing the pre-culture medium YPD (growth_medium_a) or SGM (growth_medium_b), and two mutually exclusive one-hots representing the pre-culture growth phase exponential (growth_phase_a) or stationary (growth_phase_b). The use of mutually exclusive one-hots was preferred since this preserves the non-ordinal nature of the input data, and prevents numerical bias where the model might weigh categories labelled with a larger number higher than those labelled with smaller numbers. Similarly, the presence of a particular yeast species was encoded in four one-hot categories for each species: *S. cerevisiae* as yeast_1_sc, *L. thermotolerans* as yeast_2_lt, *T. delbrueckii* as yeast_3_td, and *W. anomalus* as yeast_4_wa. Time was represented as a timestamp in hours and percentage abundance of the yeast species in multispecies culture was represented as a value between 0 and 1, with *S. cerevisiae* as yeast_1_abundance, *L. thermotolerans* as yeast_2_abundance, *T. delbrueckii* as yeast_3_abundance, and *W. anomalus* as yeast_4_adundance. Absolute cell numbers were identically represented, with column names indicating ‘absolute’. Biological repeat values are given for each data row.

##### GBR model generation

The next step was to use the pre-processed data as the basis for generating a GBR model. For this, it is necessary to classify the variables in the dataframe as features or targets. Features refer to the input variables of the model, while targets are the output values that the model is trained to predict based on the input features.

The dataset was read into a Python environment using Pandas (version 1.2.4) in a Google Collaboratory notebook (Supplementary Code S1). The dataset contained 13 features, namely time, growth medium (growth_medium_a: YPD, growth_medium_b: SGM), growth phase (growth_phase_a: exponential, growth_phase_b: stationary), a binary indicator for presence of each yeast species at inoculation (yeast_1_sc: *S. cerevisiae*, yeast_2_lt: *L. thermotolerans*, yeast_3_td: *T. delbrueckii*, yeast_4_wa: *W. anomalus*), and initial abundance of each yeast species at inoculation (yeast_1_initial_abundance → yeast_4_initial_abundance). The targets of the model were the relative abundance or absolute abundance of each yeast species (abundance: yeast_1_abundance → yeast_4_abundance, absolute: yeast_1_absolute → yeast_4_absolute). The choice to include both relative and absolute abundance as targets was made since each of these parameters provides a unique view of the dynamics of the community. Relative abundance is key for showing the composition and temporal shifts within microbial communities, while absolute abundance provides perspective on the actual scale of microbial presence and activity. Since absolute abundance quantifies the total number of microbial cells or the overall biomass in a specified environment, it provides a measure of the scale at which each yeast species population exerts ecological functions. Combining both methods yields a more accurate and comprehensive understanding of the system (Props et al. 2017, Morton et al. 2019).

Next, it is necessary to split the total dataframe into rows that will be used during training of the model and rows that will be excluded or hidden during training and used for testing the accuracy of the model predictions. During these tests, for the rows that are excluded during training, the model will provide a prediction for what the target values are, and this prediction is compared to the actual target data within that row, and this comparison is used to calculate regression metrics that give an indication of model performance.

Here, the dataset was divided into training and testing sets using an 80:20 ratio using the ‘train_test_split’ function from sklearn.model_selection module (sklearn version 0.24.2). A Gradient Boosting Regressor model (from sklearn.ensemble module) was used for the prediction of target variables. The model's hyperparameters were fine-tuned using a grid search approach (GridSearchCV from sklearn.model_selection module) with a 3-fold cross-validation on the training data. The grid search explored various combinations of the following hyperparameters: ‘n_estimators’ (50, 100, 200), ‘max_depth’ (3, 4, 5), and ‘learning_rate’ (0.01, 0.1, 0.2). The ‘n_estimators’ parameter determines the number of boosting stages, balancing accuracy and overfitting risks. ‘max_depth’, indicating the depth of each tree, was varied to optimize the complexity and overfitting trade-off. The ‘learning_rate’ influences the step size in corrections, guiding the search for a balance between training duration and model efficiency. The grid search aimed to minimize the mean squared error (MSE), calculated using ‘mean_squared_error’ from sklearn.metrics module. Optimization for the number of boosting stages to perform, the maximum depth of regression estimators, and the learning rate was performed using a simple grid search since this was sufficient to obtain final models with good performance metrics.

For each target variable in the ‘absolute’ and ‘abundance’ sets, the trained model's predictions were evaluated on the held-out test dataset. Negative predictions were set to zero, ensuring meaningful interpretation using numpy’s maximum function (numpy version 1.20.1). Model performance was assessed using Concordance Correlation Coefficient (CCC), root mean squared error (RMSE), and mean absolute error (MAE)—computed using respective functions from sklearn.metrics module. The use of CCC is important to note since this metric is amenable to compositional data and provides an indication of both precision and accuracy of the models, allowing for comparisons to be made across all the models generated here.

##### Model analysis and visualization

An important aspect of ML-based model analysis is interpretability. Here, feature importance analysis was carried out, which quantifies the contribution of each input parameter (feature) to the accuracy of the generated model, thereby giving a score of importance between 0 and 1. A higher feature importance score indicates that the particular input parameter plays a bigger role in the model’s predictions. Importantly, the magnitude of a feature importance score can be correlated to the influence on the model’s predictions, but it does not provide information about the direction of the effect or concrete evidence of any causal relationship. Still, feature importance scores are highly useful in improving interpretation of a model and provide important insights into the underlying processes of the model, which researchers can take advantage of when analysing complex datasets with several variables, such as those commonly found in microbial ecology datasets.

Feature importance values were calculated and plotted to understand which features contributed most to the model's predictions. The data visualization was carried out using Matplotlib (version 3.4.2) and GraphPad Prism 9. A threshold for feature importance value significance was calculated based on the point where the first derivative of the slope of the cumulative feature importance curve for a particular model falls below a threshold of 0.01, and increases in cumulative importance diminish significantly (Supplementary Fig. S5). Simply put, when an additional feature increased the cumulative feature importance by <0.01, indicating that adding more features beyond this point yields diminishing returns, it was disregarded.

Additionally, as provided in the supplementary materials, learning curves were plotted to illustrate the effect of the training set size on the model’s performance (Supplementary Fig. S3), and predicted vs. actual value plots were created (Supplementary Fig. S4) to visualize the model’s prediction accuracy for each target variable.

#### Subset community datasets as input

Here, the dataset was subset based on community complexity, and for each subset, the processes outlined in sections 2.6.1.1–2.6.1.3 were repeated. In the ‘pair-based’ subset, data exclusively comprising pairs of yeast species (*n* = 2) was isolated (Supplementary Dataset File, Supplementary Table S1, Supplementary Figs S6, S7). The ‘community-based’ subset encompassed data involving more than two yeast species (*n* > 2) in multi-species cultures (Supplementary Dataset File, Supplementary Table S2, Supplementary Fig, S8, S9). Based on each subset as input, respective models were trained and validated (Supplementary Codes S2 and S3). This enabled targeted analyses of various community configurations, to gain insights into the dynamics of yeast interactions under distinct conditions.

#### Application of the model generation and interpretation approach to a published dataset

The model generation and performance evaluation procedure were repeated for the dataset reported in Bagheri et al. 2020 (Supplementary Dataset File, Supplementary Code S4, Supplementary Table S3, Supplementary Figs S12, S13), where the following was specific to this dataset.

The dataset contained 19 features, namely time (represented as a value between 0 and 1 for the beginning, middle and end of fermentation), temperature (temperature_a: 15⁰C and temperature_b: 25⁰C), growth medium (medium_a: SGM, medium_b: Chenin blanc grape must, and medium_c: Grechetto bianco grape must), sulphur addition (sulphur_low: 0 mg/l and sulphur_high: 30 mg/l), a binary indicator variable for *S. cerevisiae* inoculation (Inoculated_sc), and initial abundance of 10 different yeast types (yeast_1_initial_abundance through yeast_10_initial_abundance), namely, in order from 1 to 10: *Metschnikowia pulcherrima, Pichia terricola, Starmerella bacillaris, Candida parapsilosis, W. anomalus, L. thermotolerans, Hanseniaspora vineae, S. cerevisiae* EC1118, *H. uvarum, S. cerevisiae* Indigenous Strain. The targets of the model were the abundance of these 10 yeast types, expressed as ‘yeast_1_abundance’ through ‘yeast_10_abundance’ through time. Since features associated with *H. uvarum* were all beneath the threshold, the results for this community member were not reported.

Since the data reported were averages, to generate more training data points, the original dataset was supplemented with 4 points of synthesized data that were randomly generated around the mean of the data points (Supplementary Code S4).

### Data and code availability

All datasets are provided in the supplementary materials. All code used is provided as Google Collaboratory Notebooks (Supplementary Code S1-S4).

## Results

### A grid-search tuned gradient boosted regression tree model accurately describes the temporal dynamics of a synthetic yeast community

A GBR model was successfully trained and optimized to describe the relative and absolute abundance dynamics of a four-species synthetic yeast community in various species combinations and pre-culture conditions. The choice of model targets, namely relative abundance and absolute abundance of species are all commonly used to describe population dynamics in microbial communities, which allows for extrapolation of this modelling approach to several different population measurement methodologies. The optimized parameters as well as the performance metrics for the optimized models are reported in Table 2. According to these metrics, the model captures the population dynamics trends within the dataset fairly well, confirming the applicability of the GBR framework for this datatype, as well as the grid-search approach for hyperparameter tuning. As such, the model algorithm of GBR was an appropriate choice for this dataset, and a high interpretation confidence can be attributed to the model parameters.

**Table 2.** Optimal parameters and performance of the GBR models.

Target population measure	Target yeast species model	Model parameter	Optimal parameter value	Model performance metric	Metric value
**Percent abundance**	*Saccharomyces cerevisiae*	n_estimators^1^	100	CCC score^4^	0.99
		max_depth^2^	5	Mean absolute error	0.03
		learning_rate^3^	0.2	Root mean square error	0.04
	*Lachancea thermotolerans*	n_estimators^1^	100	CCC score^4^	0.99
		max_depth^2^	5	Mean absolute error	0.02
		learning_rate^3^	0.1	Root mean square error	0.04
	*Torulaspora delbrueckii*	n_estimators^1^	200	CCC score	0.98
		max_depth^2^	4	Mean absolute error	0.02
		learning_rate^3^	0.1	Root mean square error	0.05
	*Wickerhamomyces anomalus*	n_estimators^1^	200	CCC score^4^	0.97
		max_depth^2^	5	Mean absolute error	0.03
		learning_rate^3^	0.1	Root mean square error	0.04
**Absolute cell numbers**	*Saccharomyces cerevisiae*	n_estimators^1^	200	CCC score^4^	0.99
**(per ml)**		max_depth^2^	5	Mean absolute error	6.50E + 06
		learning_rate^3^	0.1	Root mean square error	9.82E + 06
	*Lachancea thermotolerans*	n_estimators^1^	50	CCC score^4^	0.94
		max_depth^2^	5	Mean absolute error	4.81E + 06
		learning_rate^3^	0.1	Root mean square error	8.99E + 06
	*Torulaspora delbrueckii*	n_estimators^1^	100	CCC score^4^	0.96
		max_depth^2^	4	Mean absolute error	4.74E + 06
		learning_rate^3^	0.1	Root mean square error	9.27E + 06
	*Wickerhamomyces anomalus*	n_estimators^1^	100	CCC score^4^	0.96
		max_depth^2^	5	Mean absolute error	3.89E + 06
		learning_rate^3^	0.1	Root mean square error	6.99E + 06

1: Number of boosting stages to perform 2: Maximum depth of the individual regression estimators 3: Shrinks the contribution of each tree by the learning rate 4: Concordance Correlation Coefficient

### Pre-culturing methodologies had minimal influence on community dynamics

**Figure 1.**
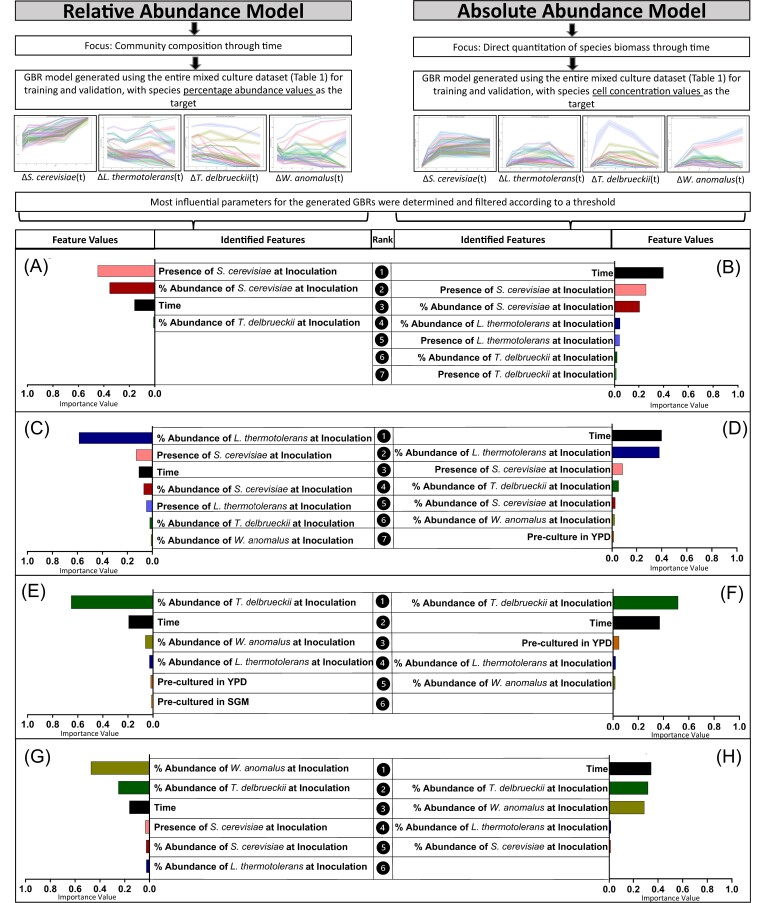
Feature importance values for GBR models trained on the full multispecies culture dataset. The model targets were either relative abundance (A, C, E, and G) or absolute abundance (B, D, F, and H). Feature values are reported for each species within the community, namely *S. cerevisiae* (A, B), *L. thermotolerans* (C, D), *T. delbrueckii* (E, F), and *W. anomalus* (G, H).

To evaluate whether the environment of each yeast prior to its introduction to the community influenced succession patterns, we made use of differing pre-culturing methodologies (Fig. 1). The pre-culture conditions tested were growth medium that the inoculums were cultured in and growth phase at which the inoculums were harvested. Overall, pre-culture had a minimal influence on the models that describe each yeast species’ succession in the community (i.e. feature importance values were below the calculated significance threshold), suggesting that community succession did in most cases not depend on the prior state of the inoculated cells (Supplementary Fig. S10). One exception, however, was *T. delbrueckii*, where the feature importance value for pre-culture in YPD was ranked third in the GBR model of absolute abundance through time (Fig. 1F, Supplementary Fig. S10E, F).

### Inoculation dosage of non-*Saccharomyces* species influences their performance during mixed culture growth, while *S. cerevisiae* performs consistently regardless of inoculation dosage

The GBR models were generated with input features that indicated the presence of a particular species (dosage-independent), as well as input features that indicated the inoculation dosage of each species, *allowing* for us to determine how predictive initial abundance is for temporal succession patterns. Interestingly, the inoculation dosage was ranked consistently high among all community members (Fig. 1), however, for *S. cerevisiae*, the dosage-independent feature (i.e. Feature: Presence of *S. cerevisiae* at inoculation) was higher ranked (Fig. 1A, B). Therefore, *S. cerevisiae* ultimately dominates the wine community through time, regardless of its initial abundance values. Also, the strongest predictor for non-*Saccharomyces* succession patterns within this community was the amount at which they were initially inoculated.

### In mixed cultures with more than two community members, the initial dosage of *S. cerevisiae* impacts *L. thermotolerans* and *W. anomalus* performance

To evaluate how succession patterns in pairwise communities may be altered by the presence of additional species, two separate GBR models were trained and validated on the relative and absolute abundance through time for respective pairwise or complex mixed culture settings. The results were consistent between the relative and absolute abundance models (Supplementary Fig. S11); therefore, for brevity, the relative abundance models are presented. The model performances were comparable to the original model, with high CCC scores (Supplementary Tables S1–S2).

**Figure 2.**
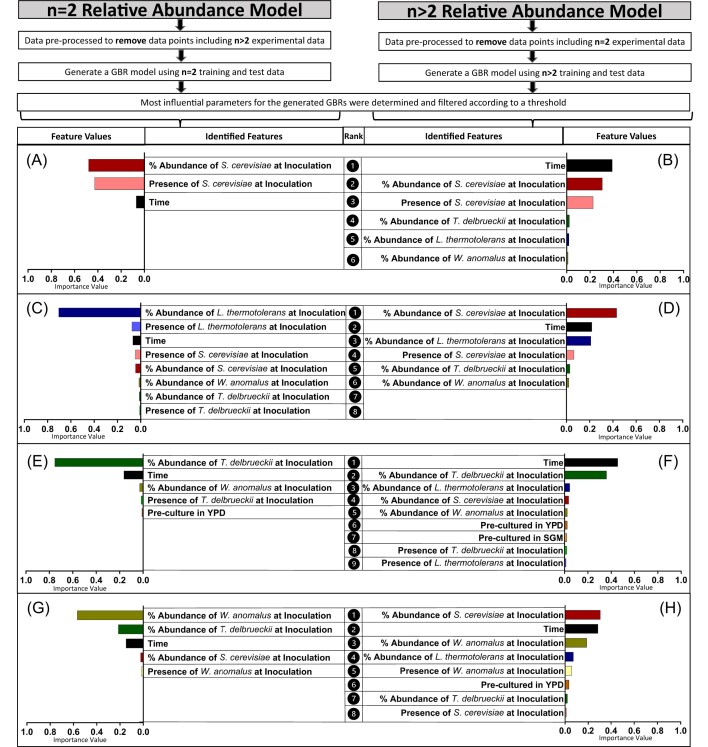
Feature importance values for GBR models trained on subsets of the dataset, including either 2 (A, C, E, G) or more than 2 (B, D, F, H) species in the multispecies culture. The model targets were all relative abundance. Feature values are reported for each species within the community, namely *S. cerevisiae* (A, B), *L. thermotolerans* (C, D), *T. delbrueckii* (E, F), and *W. anomalus* (G, H).

**Figure 3.**
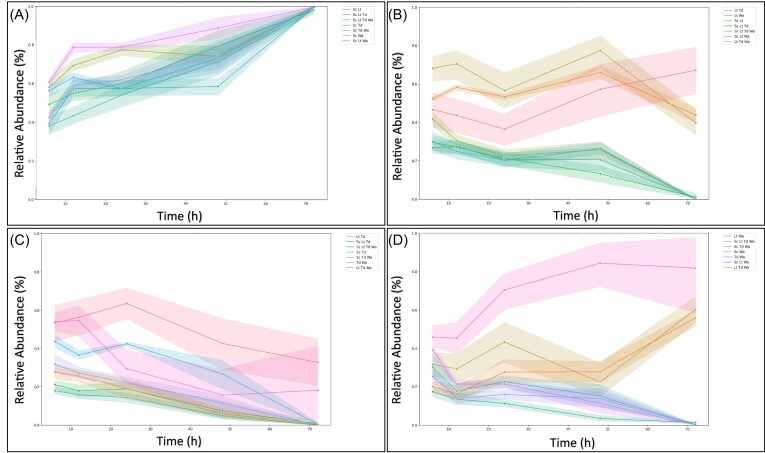
Relative abundance time series of each community member in each possible multispecies configuration tested. Values are averaged across all pre-culture conditions tested. Time series are shown for *S. cerevisiae* (A), *L. thermotolerans* (B), *T. delbrueckii* (C), and *W. anomalus* (D). The legend contains abbreviations for the particular combination of yeasts within the time series, namely Sc: *S. cerevisiae*, Lt: *L. thermotolerans*, Td: *T. delbrueckii*, and Wa: *W. anomalus*.

For *S. cerevisiae* and *T. delbrueckii*, the most important model features were relatively consistent between pairwise and more complex combinations (Fig. 2A, B, E, F), while some interesting differences were seen for the other non-*Saccharomyces* species (Fig. 2C, D, G, H). For *L. thermotolerans* and *W. anomalus*, in 3- and 4-species settings, *S. cerevisiae*. inoculation dosage is highest ranked (Fig. 2D, H), in contrast to pairwise settings (Fig. 2C, G). Exploring this further, the time-series plots of *T. delbrueckii, L. thermotolerans*, and *W. anomalus*, in various mixed culture settings, echo these features (Fig. 3B, C, D). Specifically, *T. delbrueckii* performs similarly across all 3- and 4-species settings, regardless of whether *S. cerevisiae* is present (Fig. 3C). In contrast, *L. thermotolerans* and *W. anomalus* both attain higher relative abundance values in 3-species settings where *S. cerevisiae* is absent (Fig. 3B, D).

### *Wickerhamomyces anomalus* abundance is impacted by *T. delbrueckii* during pairwise culture, while *L. thermotolerans* abundance is impacted by *S. cerevisiae* across all mixed cultures

The GBR model features also highlighted species-specific impacts of initial dosage on temporal succession in the synthetic communities. Simply put, this relates to how the relative amount of a particular species at inoculation may distinctly impact on another yeast species’ population dynamics. Here, the presence and dosage of *S. cerevisiae* were influential in the performance of *L. thermotolerans* in all settings (Fig. 1C, D and Fig. 2C, D), while for *W. anomalus, T. delbrueckii* was influential during pairwise growth, but this strong pairwise interaction was not retained when additional species were present (Fig. 2G, H). For *S. cerevisiae*, performance was largely consistent regardless of what culture setting it was exposed to (Fig. 2A, B and Fig. 3A), and *T. delbrueckii* was influenced most by its initial inoculation dosage (Fig. 2C, D).

### The GBR model generation and interpretation approach is effective across different yeast consortia and environments

To further test the applicability of GBR models for highlighting factors that impact succession, we analysed a published dataset of a more complex wine yeast community in a more industrially relevant fermentation environment.

**Figure 4.**
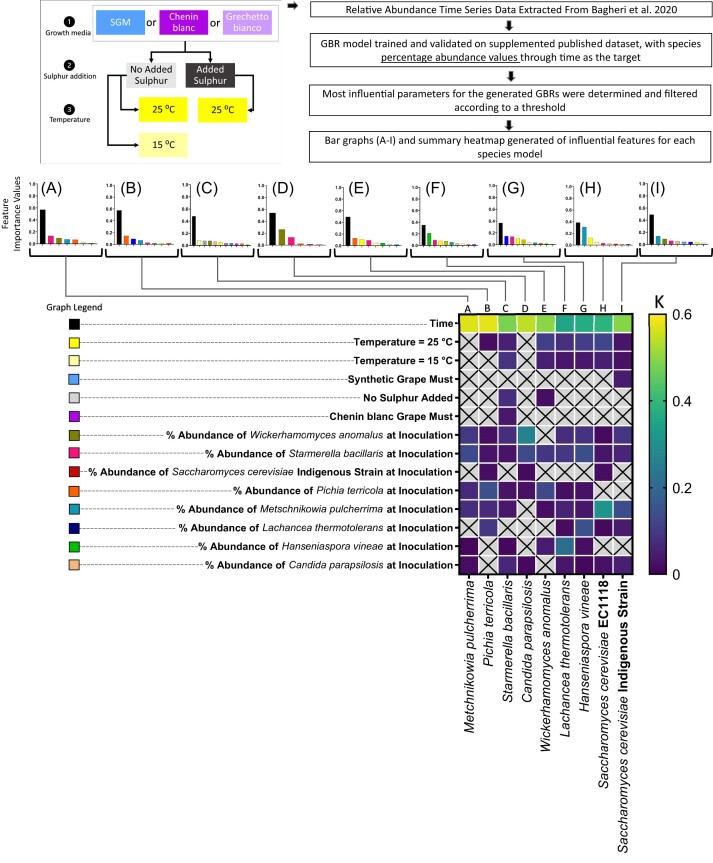
Feature importance values for GBR models trained on relative abundance of a nine-species wine yeast community in different growth media, growth temperatures, and sulphur addition. Feature importance values for each species are reported, namely: *M. pulcherrima* (A), *P. terricola* (B), *S. bacillaris* (C), *C. parapsilosis* (D), *W. anomalus* (E), *L. thermotolerans* (F), *H. vineae* (G), *S. cerevisiae* EC1118 (H), and *S. cerevisiae* Indigenous Strain (I). The legend for A–I is provided, with each bar colour representing a model feature. A summary heatmap of the feature importance values is provided for comparison, with a colour scale where darker blue is a low value and brighter yellow is a higher value (K).

Here, 10 potential community members were grown in three different growth media, and under different temperature and sulphur concentrations, and community succession was monitored throughout fermentation (Fig. 4). This dataset was generated as a comparative investigation into the impact of abiotic and biotic factors on metabolic and community outcomes, and is a prime example of a multi-variate, multi-species dataset, for which data interpretation in a quantifiable way would be challenging.

The GBR model successfully described community dynamics, with high CCC scores (Supplementary Table S3, Supplementary Fig. S13). Here, we compared the insights generated by our models to the results that were originally reported by Bagheri et al. as an illustration into what additional insights our approach provides for understanding the community succession within this complex dataset. The feature importance analysis of the resultant GBR models indeed captured the broad results that were originally reported by Bagheri et al. namely that the most influential input parameters were biotic in nature, while the abiotic factors: growth medium and sulphur concentration were less influential (Fig. 4).

Specifically, our analysis showed low or non-existent influence of growth media and sulphur addition parameters, and high influence of inoculation dosage of community members (Fig. 4A–I). Interestingly, temperature was highlighted as an influential parameter within this community, being ranked within the top four most influential model input features for *S. bacillarus* (Fig. 4C), *W. anomalus* (Fig. 4E), *L. thermotolerans* (Fig. 4F), *H. vineae* (Fig. 4G), and *S. cerevisiae* EC1118 (Fig. 4H).

Similar to the results within our synthetic community, this analysis also highlighted specific impacts of initial abundance of certain community members on each other. Namely, the initial abundance of *M. pulcherrima* ranked highly for impacting temporal dynamics of both *S. cerevisiae* strains (Fig. 4H, I), the initial abundance of *W. anomalus* appeared to impact *C. parapsilosis* performance within the community (Fig. 4D), and finally, there was a reciprocal impact of initial abundance of *L. thermotolerans* and *H. vineae* on each other’s succession within the community (Fig. 4F, G). These results provide clues into how this complex community may be manipulated, and which factors are most important in controlling the wine yeast community.

## Discussion

This study aimed to shed light on how factors pertinent to arrival or inoculation impact on community succession within a model yeast ecosystem. By modelling population dynamics with a ML algorithm, we sought to investigate how a yeast’s environment prior to inoculation, as well as initial abundance at inoculation impact on the trajectory of each yeast. A comprehensive dataset of temporal community data was generated in various numbers and combinations of wine yeast species, as well as differing preparation methods for each species’ inoculum. By leveraging GBR models, we identified the most likely factors related to inoculation that alter temporal dynamics of each community member, and validate the strength of this approach in a previously published dataset that includes more varied environmental parameters.

The first research question related to how the history of a particular yeast species prior to its arrival in the community would impact on its trajectory within a mixed-species culture. In the four-species yeast community investigated here, differing pre-culture conditions impacted far less than the combination of yeast species present, except for *T. delbrueckii*. This may be linked to previously observed high stress response observed in the transcriptome of *T. delbrueckii* during adaptation to a high sugar medium (Tondini et al. 2020). This highlights the challenge that even for studies using synthetic systems with reduced complexity, a careful evaluation of pre-culture conditions should be carried out, as species-specific impacts may arise.

The second research question related to how inoculation dosage, or relative abundance at arrival into the community, impacted on community succession. The initial absolute abundance of yeasts present at inoculation impacted the performance of *L. thermotolerans, T. delbrueckii*, and *W. anomalus* in pairwise cultures; however, in 3- and 4-species cultures, the initial abundance of *S. cerevisiae* was most impactful for *L. thermotolerans* and *W. anomalus*. In contrast, *S. cerevisiae*’s temporal dynamics were relatively consistent and independent of inoculation dosage, in line with previous studies that have shown the dominance of *S. cerevisiae* in later stages of fermentation despite comprising a very small part of the total yeast population at the start of fermentation (Combina et al. 2005, Bagheri et al. 2017, Granchi et al. 2019, Conacher et al. 2021). The relative abundance of *L. thermotolerans* was consistently impacted by *S. cerevisiae*, and the inter-species interaction between these two yeasts is indeed well studied and is hypothesized to be antagonistic in nature (Shekhawat et al. 2019, Luyt et al. 2021). The important influence of inoculation dosage in regulating temporal dynamics has been observed in other wine yeast communities (Bagheri et al. 2018), and is an indication that this is important to consider in the quest to rationally control temporal succession in these communities.

The third research question related to how the number and identity of species present at arrival may impact on community succession, with a focus on whether we could identify signatures of multi-species-interactions in our dataset. Here we identify a strong influence of *T. delbrueckii* on *W. anomalus* in pairwise co-culture; however, the presence of *S. cerevisiae* modulates this influence. This shows that the presence of an additional species can impact dynamics of this pair in a species-specific manner. This has implications for future studies that wish to elucidate the mechanisms of interspecies interactions within the wine yeast community, as building predictive models of the system will require inclusion of data from complex multispecies cultures, and not pairwise co-cultures alone (Chang et al. 2023).

Finally, to test whether our approach is effective across datasets and to gain a better understanding of how general the influence of inoculation dosage is for yeast communities in more naturally relevant conditions, we then generated a model for a more complex wine yeast community, where abundance dynamics were measured in different temperatures, sulphur concentrations, and growth media—including real grape must (Bagheri et al. 2020). The GBR feature importance values aligned well to the originally reported analysis of the dataset, but also provided new insights, namely into the fact that inoculation dosage was also highly influential in temporal population dynamics within the community, and that temperature (but not growth medium nor sulphur addition) modulated community trends. This provides more robust evidence for our claim that inoculation dosages are highly important in determining yeast community dynamics, and introduces temperature as a way to manipulate community succession. We further also highlighted several interesting impacts between yeast species in this community that were not originally reported, namely between *M. pulcherrima* initial abundance and both *S. cerevisiae* strains—this pair has indeed been shown previously to interact with one another at the cellular and transcriptional level (Contreras et al. 2015, Mencher et al. 2021). Further interactions between the lesser-studied non-*Saccharomyces* yeasts were also highlighted. First, *W. anomalus* inoculation dosage impacted on *C. parapsilosis*, and previous studies have shown the activity of *W. anomalus* killer toxins on various *Candida* spp. (Johannes et al. 2014, de Ullivarri et al. 2020). Second, *L. thermotolerans* and *H. vineae* inoculation dosage impacted on each other, and previous studies have indeed reported that *H. vineae* modulates lactic acid production in *L. thermotolerans*, and *H. vineae* growth is impacted by the presence of *L. thermotolerans* (Vaquero et al. 2021).

We therefore provide further evidence of the strength of tree-based GBR algorithms in modelling wine yeast community-related datasets, which is in line with the success of other tree-based algorithms that have been applied in a microbial ecology context (DiMucci et al. 2018, Thompson et al. 2019, Nestor et al. 2023), and that this approach produced accurate and interpretable models across different consortia complexities and environments. This approach allowed us to quantitatively discern patterns in each dataset that may not have been readily apparent through manual inspection of the dataset or simple graphing techniques. Promisingly, the model features identified as important aligned with previously reported research findings, while still providing novel insights from the dataset that had been missed, adding credibility to this approach in the context of wrangling complex temporal datasets to mine for factors important in community succession. However, the concept of using an interpretable model to assist in identifying driving factors in multi-species systems is important to distinguish from the traditional approach of generating a model for generalizing across different datasets and simulating experiments. The approach presented here is designed as a tool for mining complex time-series datasets as they are, and the models generated are not appropriate for use as a predictive simulation tool across novel experimental conditions or datasets. Previous studies have also observed that this type of model is not accurate in providing predictions for conditions that the model has not been exposed to (DiMucci et al. 2018, Nestor et al. 2023). We also emphasize that the feature importance analysis is not causative, and is reliant on the categorical variables provided; we therefore strongly encourage further experimental validation of these features.

This study provides a significant contribution to addressing the current challenges in understanding the dynamics of microbial communities by presenting a way to quantify how species are differentially impacted by biotic and abiotic parameters within synthetic or natural ecological contexts, opening avenues for rational manipulation and mechanistic studies in targeted environments. This study is one of very few that has incorporated experimental data generation with ML for the study of a complex microbial community. Further, we provide a rare look at how a stepwise increase in complexity of a microbial community changes its succession dynamics. Notably, viable cell numbers are provided, which are usually not possible to report due to the molecular nature of most microbial community quantitation techniques. Within this context, the study is limited by the fact that it does not evaluate molecular interaction mechanisms and is based at the population-cellular level. Also, incorporation of more experimental abiotic parameters may have shown that factors besides inoculation dosage, such as oxygenation or available nitrogen, may be more impactful in modulating community succession, and we recommend exploring this further with the aid of the approach we present here. In addition, it will be essential to link the manipulation of temporal succession dynamics to community functioning, such as sugar consumption, in future studies. Further, exploring more complex ML tools that incorporate time series memory, such as recurrent neural networks, may improve generalizability (Thompson et al. 2023).

We envision that the concepts and data presented here should aid in bridging the gap in understanding complex dynamics in microbial communities by accelerating data interpretation and allowing for targeted experimental design. This should also significantly aid in the challenge of rational design of synthetic consortia, since community succession is an important factor in the functioning of microbial communities. With the resources provided in this manuscript, particularly for non-experts in coding, reproducing model generation for any target dataset should be possible within reason. Given the rapid pace of integration of ML algorithms into microbiological research, this work presents an approachable and powerful analysis tool for domain experts to get the most out of their microbial community datasets.

## Supplementary Material

fiae080_Supplemental_Files
