# Supplementary material for: Gradient boosted regression as a tool to reveal key drivers of temporal dynamics in a synthetic yeast community
Source: FEMS Microbiol Ecol. 2024 May 22;100(7):fiae080. doi: 10.1093/femsec/fiae080 (PMC11212668; doi:10.1093/femsec/fiae080)
Supplement: fiae080_Supplemental_Files [file fiae080_supplemental_files.zip › FEMS ME Supplementary Materials Revised.docx]

**Supplementary Material for: Gradient boosted regression as a tool to reveal key temporal drivers in a synthetic yeast community**

Conacher CG*^a,b^, Watson BW^b^, Bauer FF*^a^

^a^ South African Grape and Wine Research Institute, Department of Oenology and Viticulture, Private Bag X1, Stellenbosch University, Stellenbosch, 7600, South Africa

^b^ Centre for AI Research, School for Data‑Science & Computational Thinking, Stellenbosch University, Stellenbosch 7600, South Africa.

(*) co-corresponding author

Author’s details:

Cleo G. Conacher*: [cleoconacher2@gmail.com](mailto:cleoconacher2@gmail.com)

Bruce W. Watson: [bwwatson@sun.ac.za](mailto:bwwatson@sun.ac.za)

Florian F. Bauer*: [fb2@sun.ac.za](mailto:fb2@sun.ac.za)

**A**

**B**

**C**

**D**


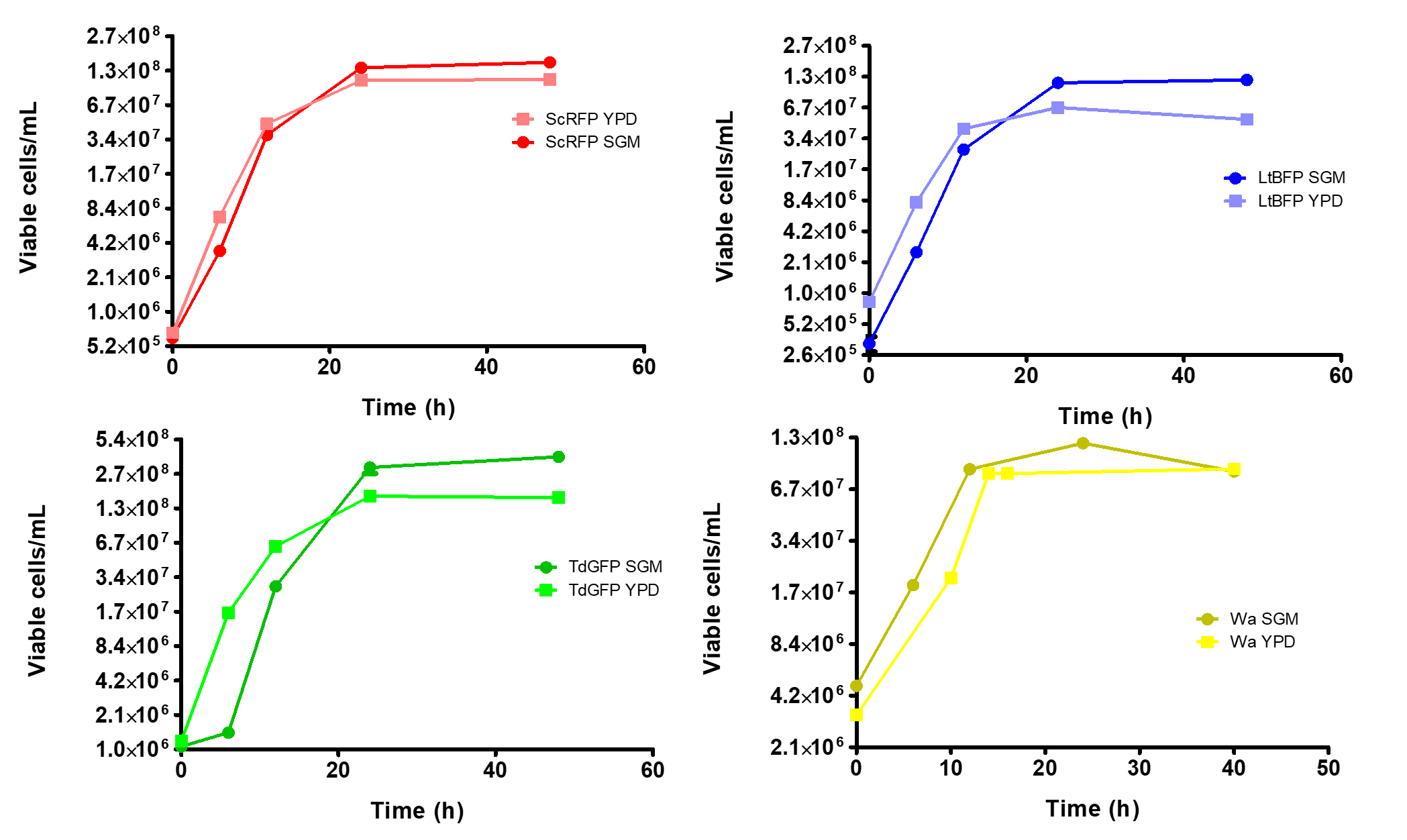


**Supp. Fig. 1.** Growth curves used to confirm pre-culture growth phase. Circles denote pre-cultures grown in Synthetic Grape Must (SGM), squares denote pre-cultures grown in Yeast Peptone Dextrose (YPD). A: *S. cerevisiae*, B: *L. thermotolerans*, C: *Torulaspora delbrueckii*, D: *Wickerhamomyces anomalus*.


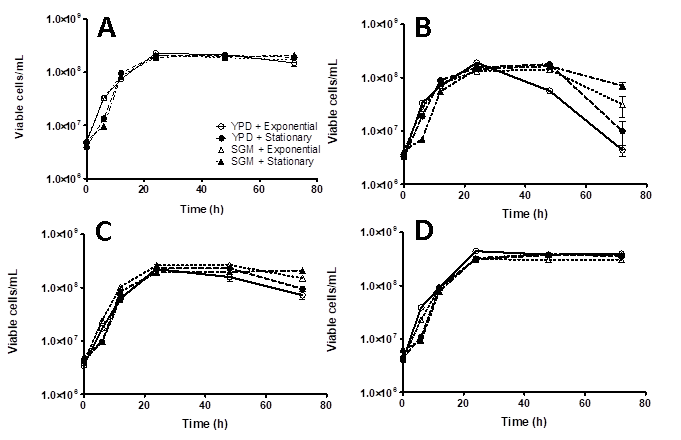


**Supp. Fig. 2.** Monoculture growth curves after using pre-cultures conducted in YPD or SGM to exponential or stationary growth phase. A: *S. cerevisiae*, B: *L. thermotolerans*, C: *T. delbrueckii*, D: *W. anomalus*. Open circles, solid line: YPD + Exponential phase. Filled circles, even long dashed line: YPD + Stationary phase. Open triangles, even short, dashed line: SGM + Exponential phase. Filled triangles, mixed dashed line: SGM + Stationary phase. Error bars represent standard error from the mean, with a minimum of two biological replicates.

**Supp. Fig. 3.** Comparative learning curves of Gradient Boosting Regressor for each target variable of the model using the complete dataset as input. Each subfigure (A-I) illustrates the learning curve for a separate target variable, with the x-axis representing the number of training examples and the y-axis denoting the model's performance metric (mean squared error). The blue line plots the performance on the training set, while the orange line denotes the performance on the validation set. The proximity of these two lines and their convergence pattern provide an indication of the model's learning progress and potential for improvement with additional data. Subfigures: (A) yeast_1_absolute, (B) yeast_2_absolute, (C) yeast_3_absolute, (D) yeast_4_absolute, (E) yeast_1_abundance, (F) yeast_2_abundance, (G) yeast_3_abundance, and (H) yeast_4_abundance.

**H**

**E**

**F**


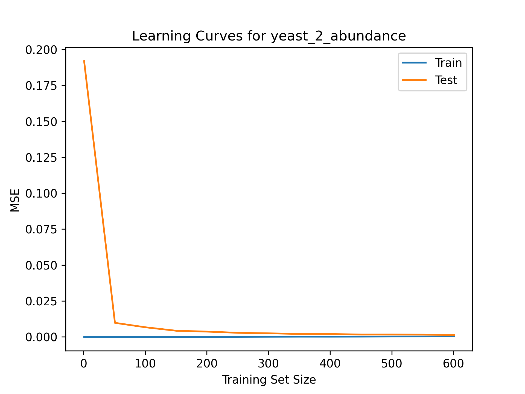

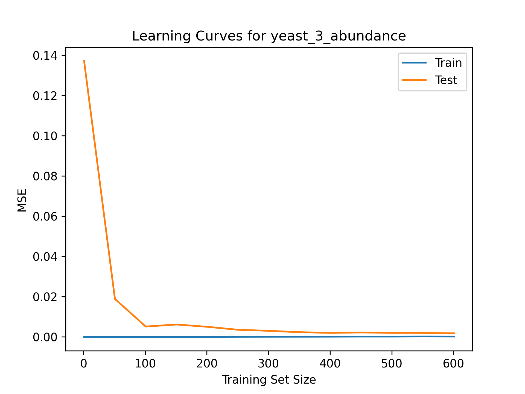

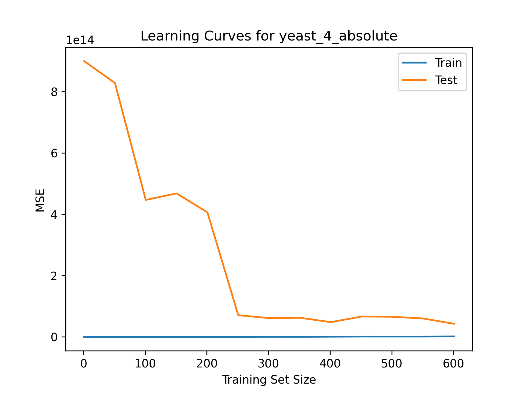

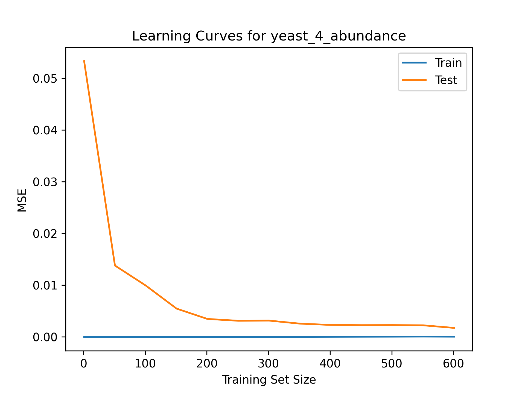

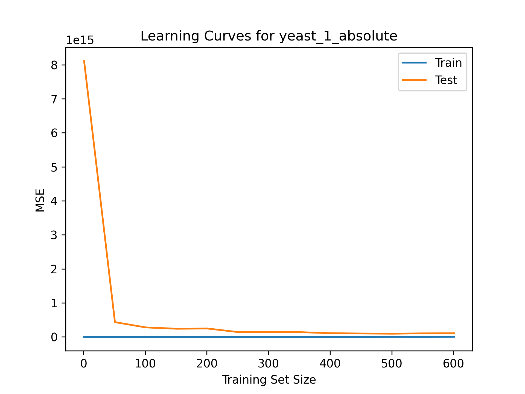

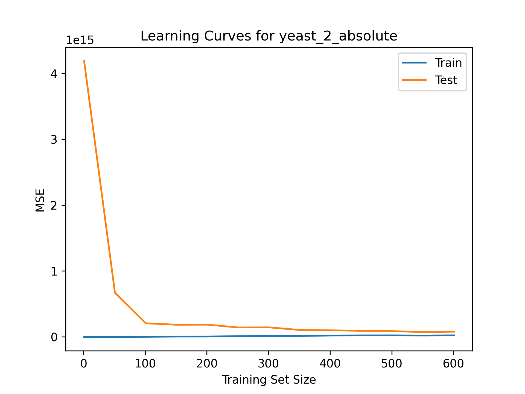

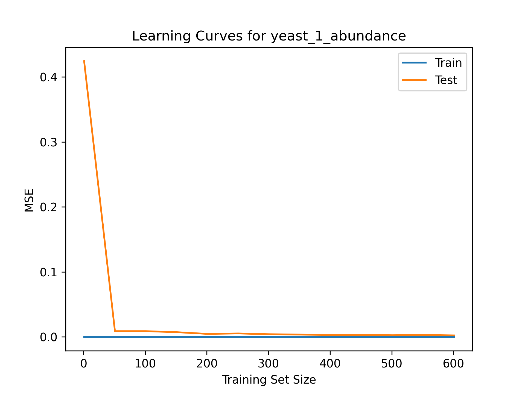

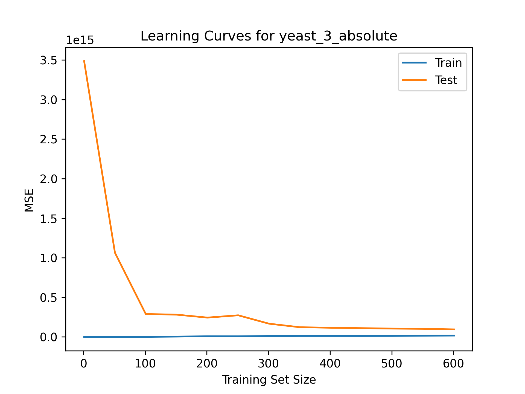


**A**

**B**

**C**

**D**

**G**

**
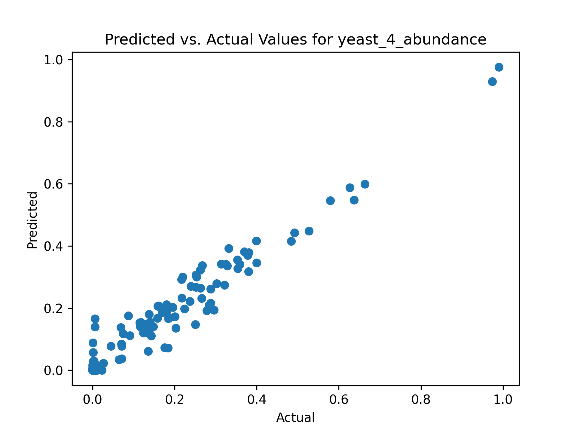

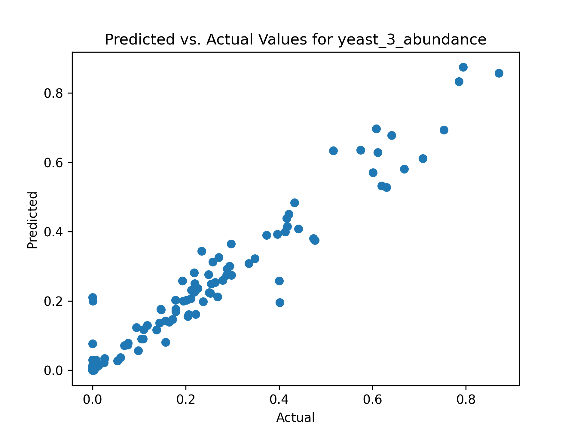

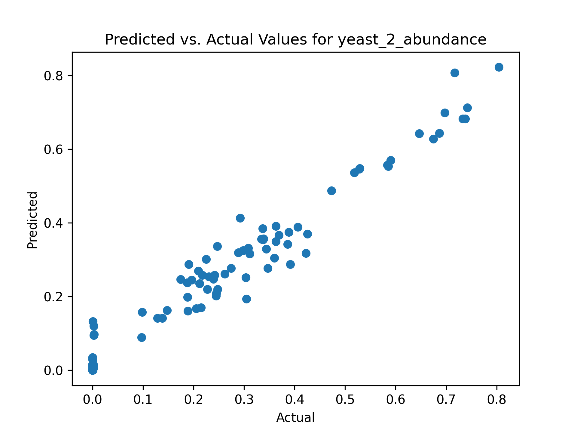

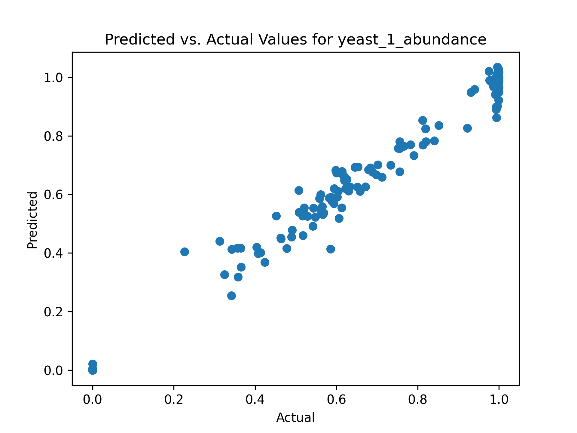

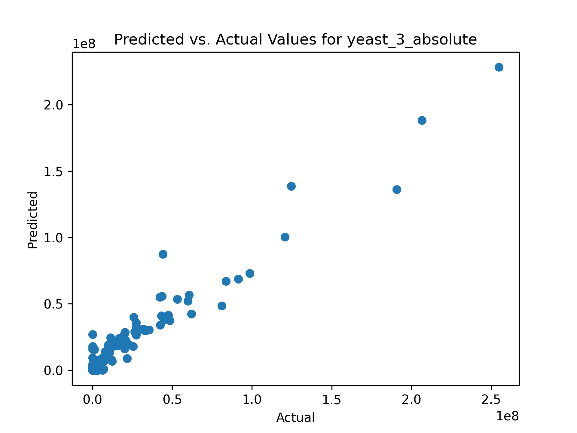

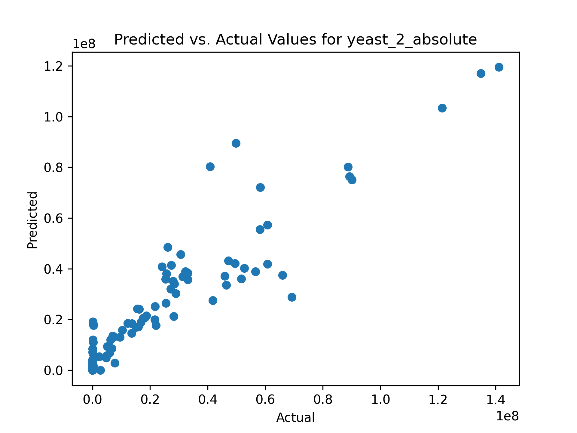

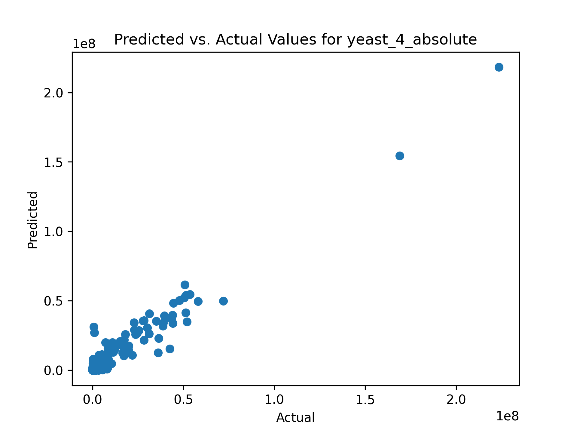

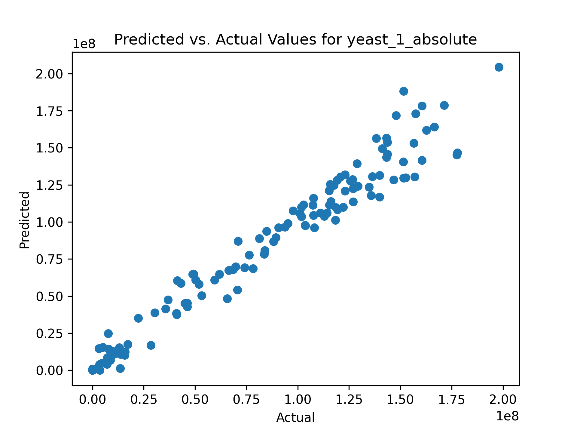
**

**G**

**D**

**B**

**C**

**F**

**H**

**E**

**A**

**Supp. Fig. S4**. Predicted versus actual value plots for each target variable in the Gradient Boosting Regressor model trained on the complete dataset. Each subfigure (A-I) shows a scatter plot where each point represents a particular sample. The x-axis denotes the actual values, and the y-axis denotes the predicted values for each target. A perfect model would result in all points aligning along the diagonal line, which represents a one-to-one correspondence between predicted and actual values. Deviations from this line indicate prediction errors. Subfigures: Subfigures: (A) yeast_1_absolute, (B) yeast_2_absolute, (C) yeast_3_absolute, (D) yeast_4_absolute, (E) yeast_1_abundance, (F) yeast_2_abundance, (G) yeast_3_abundance, and (H) yeast_4_abundance.


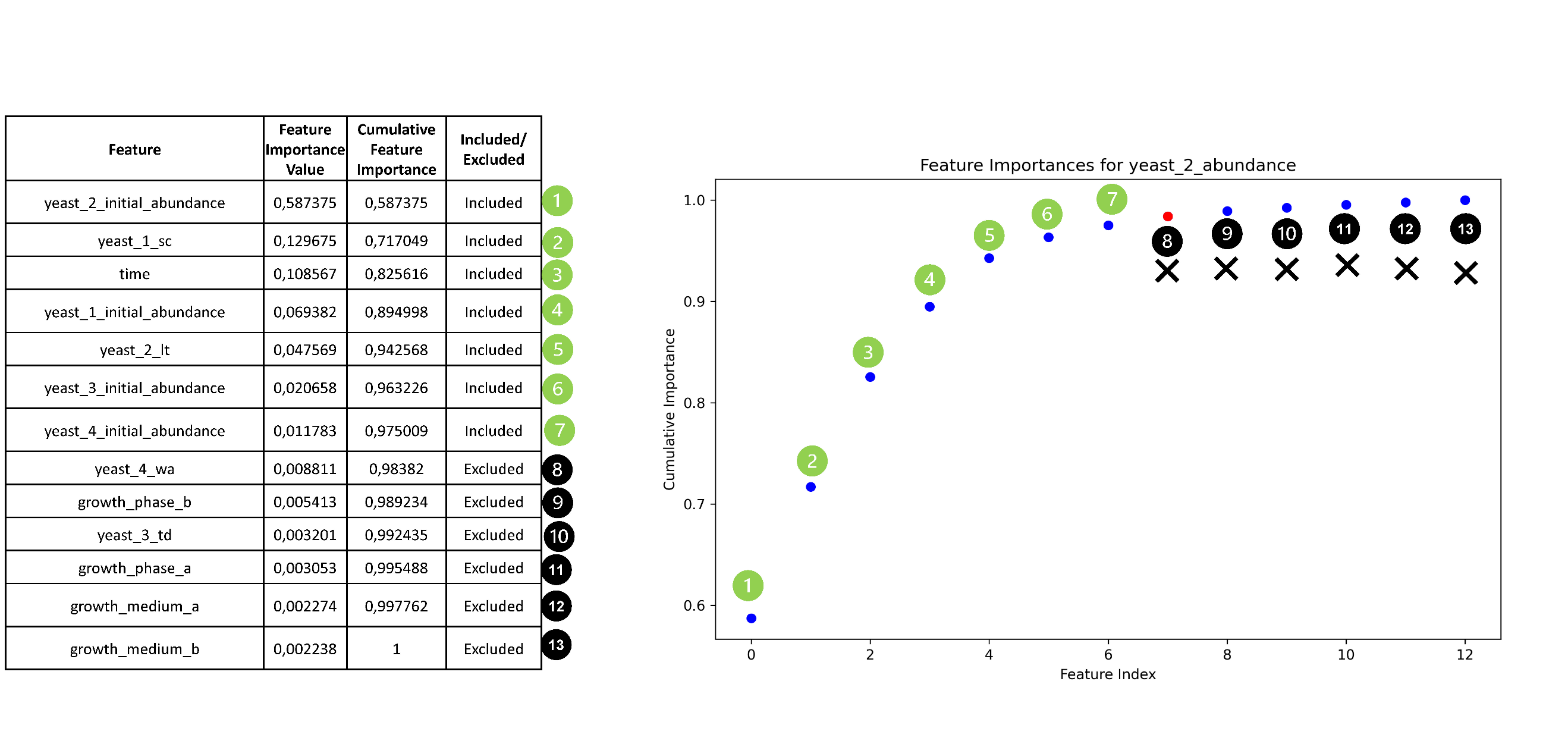


**Supp. Fig. S5**. Example of the thresholding applied during feature importance analysis. Feature importance values for *Lachancea thermotolerans* abundance in the complete GBR model are shown. A threshold for feature importance value significance was calculated based on the point where the first derivative of the slope of the cumulative feature importance curve for a particular model falls below a threshold of 0.01, and increases in cumulative importance diminish significantly.

**Supp. Table 1.** Model metrics for pair-based GBR model.

| Target | Best Parameters | MAE | CCC | RMSE |
| --- | --- | --- | --- | --- |
| yeast_1_absolute | {'learning_rate': 0.1, 'max_depth': 4, 'n_estimators': 100} | 5266664.008306008 | 0.9903109261239499 | 9442967.439970559 |
| yeast_2_absolute | {'learning_rate': 0.1, 'max_depth': 4, 'n_estimators': 50} | 4504858.03123609 | 0.964502269018404 | 9375317.556559809 |
| yeast_3_absolute | {'learning_rate': 0.2, 'max_depth': 3, 'n_estimators': 50} | 7049622.959963111 | 0.9253762821637984 | 12211419.31412872 |
| yeast_4_absolute | {'learning_rate': 0.1, 'max_depth': 5, 'n_estimators': 50} | 3937186.4994910667 | 0.9824898776403463 | 8189545.22621458 |
| yeast_1_abundance | {'learning_rate': 0.1, 'max_depth': 5, 'n_estimators': 100} | 0.01440632907701652 | 0.9977328558287587 | 0.026472544012308325 |
| yeast_2_abundance | {'learning_rate': 0.1, 'max_depth': 5, 'n_estimators': 50} | 0.02181417120441597 | 0.9851779167095748 | 0.04522477799135569 |
| yeast_3_abundance | {'learning_rate': 0.2, 'max_depth': 3, 'n_estimators': 100} | 0.03139341050030518 | 0.9784084177240099 | 0.051284690718189745 |
| yeast_4_abundance | {'learning_rate': 0.2, 'max_depth': 5, 'n_estimators': 50} | 0.02438458421201071 | 0.983843097277478 | 0.04904729717336009 |

Yeast_1: *S. cerevisiae,* Yeast_2: *L. thermotolerans*, Yeast_3: *T. delbrueckii*, Yeast_4: *W. anomalus*


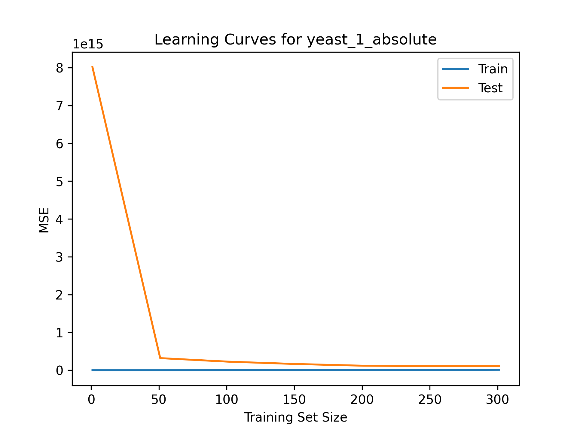

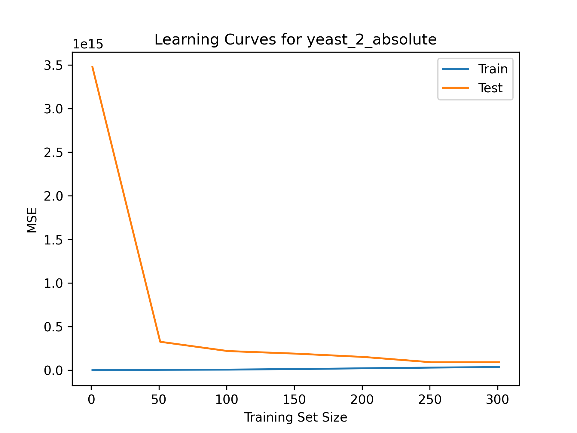

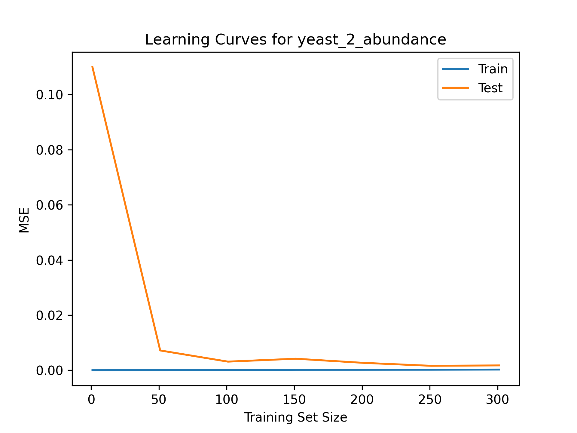

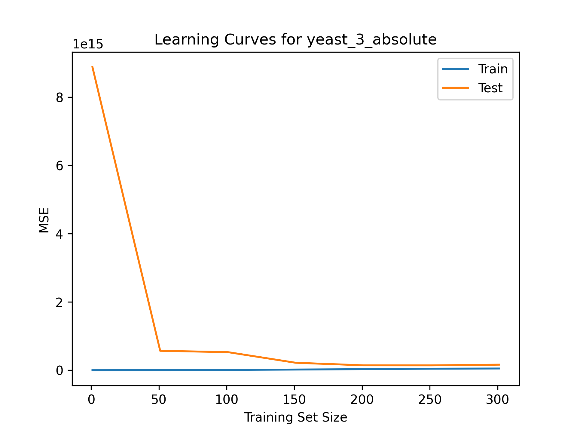

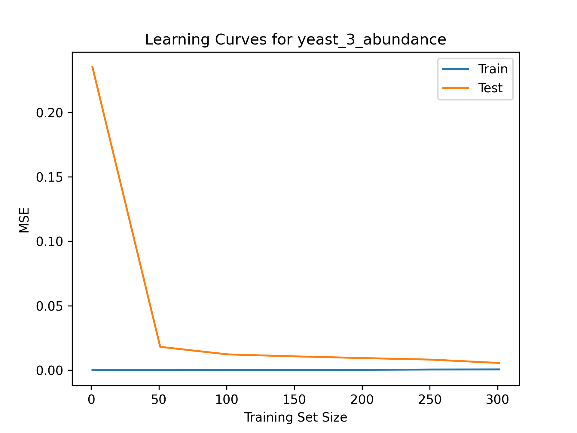

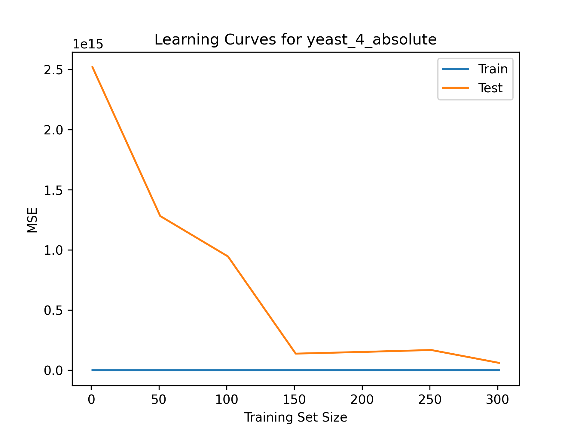

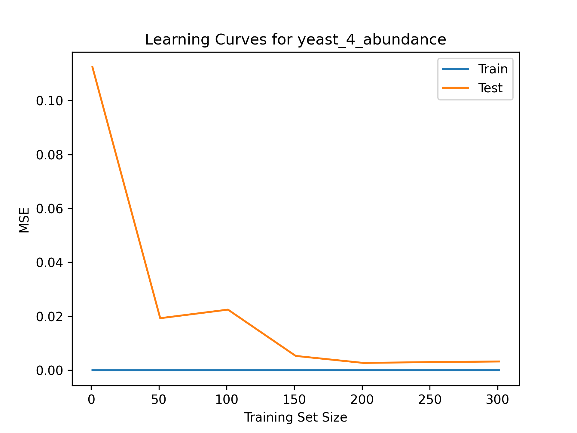

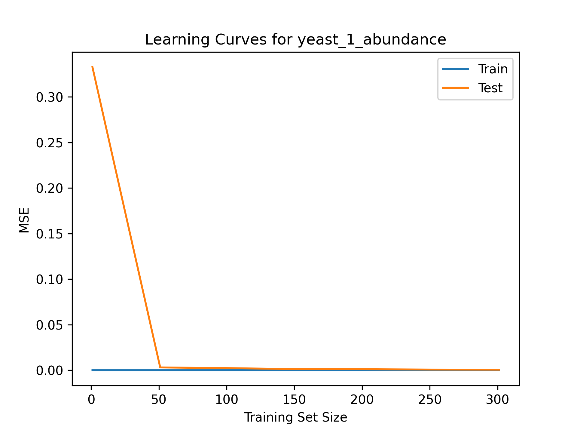


**A**

**B**

**C**

**D**

**E**

**F**

**G**

**H**

**Supp. Fig. 6.** Comparative learning curves of Gradient Boosting Regressor for each target variable of the model trained on the pair-data subset. Each subfigure (A-I) illustrates the learning curve for a separate target variable, with the x-axis representing the number of training examples and the y-axis denoting the model's performance metric (mean squared error). The blue line plots the performance on the training set, while the orange line denotes the performance on the validation set. The proximity of these two lines and their convergence pattern provide an indication of the model's learning progress and potential for improvement with additional data. Subfigures: (A) yeast_1_absolute, (B) yeast_2_absolute, (C) yeast_3_absolute, (D) yeast_4_absolute, (E) yeast_1_abundance, (F) yeast_2_abundance, (G) yeast_3_abundance, and (H) yeast_4_abundance.

**
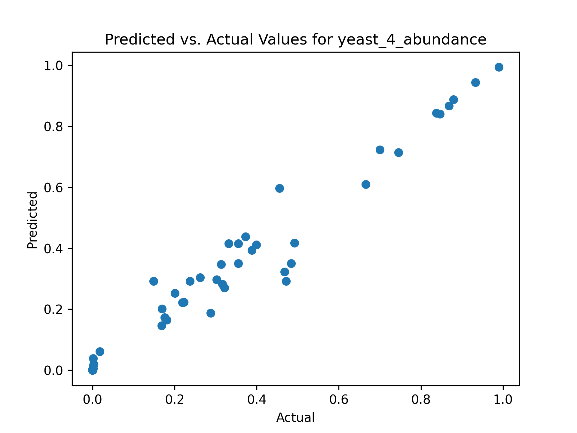

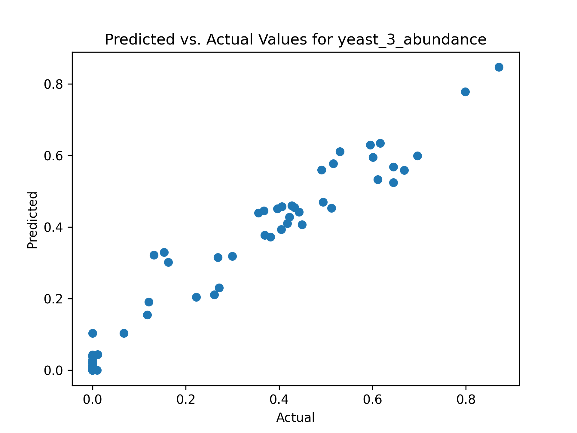

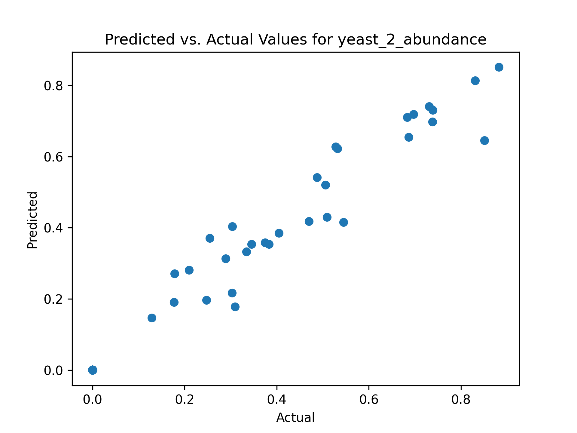

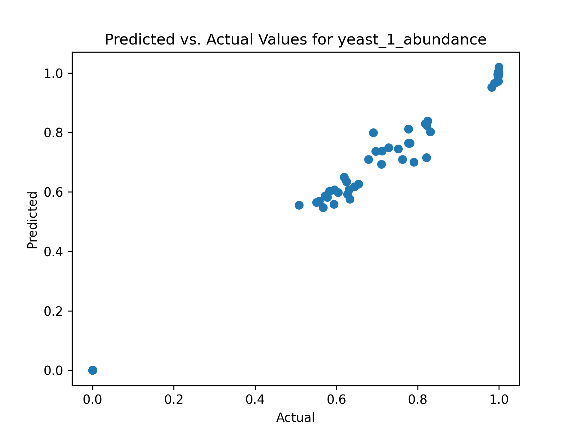
**

**
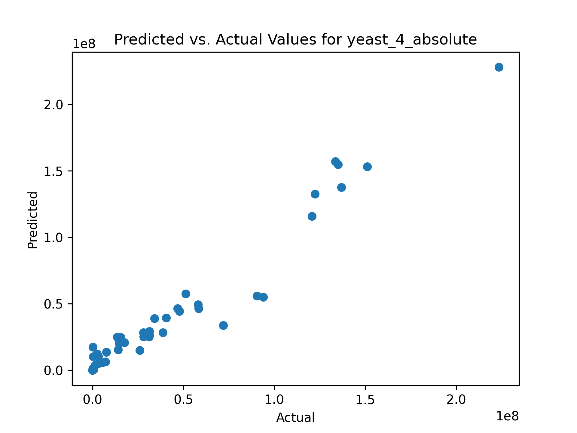

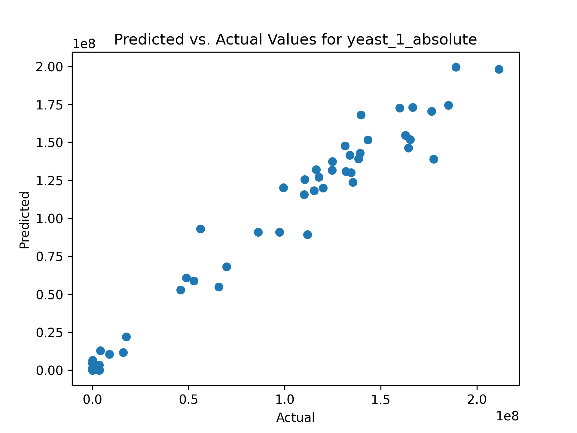

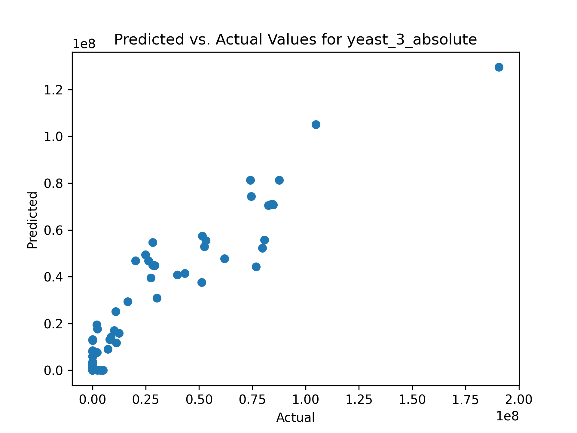

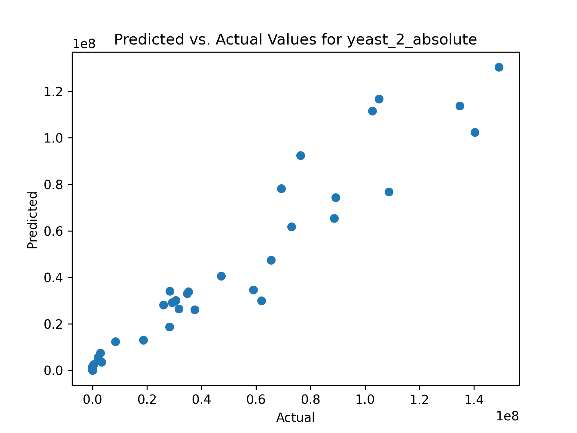
**

**E**

**A**

**H**

**G**

**F**

**D**

**C**

**B**

**Supp. Fig. 7**. Predicted versus actual value plots for each target variable in the Gradient Boosting Regressor model trained on the pair-data subset. Each subfigure (A-I) shows a scatter plot where each point represents a particular sample. The x-axis denotes the actual values, and the y-axis denotes the predicted values for each target. A perfect model would result in all points aligning along the diagonal line, which represents a one-to-one correspondence between predicted and actual values. Deviations from this line indicate prediction errors. Subfigures: (A) yeast_1_absolute, (B) yeast_2_absolute, (C) yeast_3_absolute, (D) yeast_4_absolute, (E) yeast_1_abundance, (F) yeast_2_abundance, (G) yeast_3_abundance, and (H) yeast_4_abundance.

**Supp. Table 2.** Model metrics for community-based GBR model.

| Target | Best Parameters | MAE | CCC | RMSE |
| --- | --- | --- | --- | --- |
| yeast_1_absolute | {'learning_rate': 0.1, 'max_depth': 5, 'n_estimators': 50} | 5589845.247788533 | 0.9826458678926454 | 8123461.244926776 |
| yeast_2_absolute | {'learning_rate': 0.1, 'max_depth': 5, 'n_estimators': 50} | 3552322.0481811804 | 0.9651811570479543 | 5994801.380344742 |
| yeast_3_absolute | {'learning_rate': 0.1, 'max_depth': 5, 'n_estimators': 100} | 2504839.183620963 | 0.9700190381028652 | 4104256.1251791464 |
| yeast_4_absolute | {'learning_rate': 0.1, 'max_depth': 4, 'n_estimators': 200} | 3167494.281557135 | 0.9251405833358556 | 5657189.627788142 |
| yeast_1_abundance | {'learning_rate': 0.1, 'max_depth': 5, 'n_estimators': 100} | 0.0353020020417953 | 0.9850065728570394 | 0.046931631999241996 |
| yeast_2_abundance | {'learning_rate': 0.2, 'max_depth': 5, 'n_estimators': 50} | 0.02120897283559221 | 0.9834917857512325 | 0.02988167055446537 |
| yeast_3_abundance | {'learning_rate': 0.1, 'max_depth': 4, 'n_estimators': 100} | 0.02054031779906985 | 0.9447907843005833 | 0.03620809077458748 |
| yeast_4_abundance | {'learning_rate': 0.1, 'max_depth': 5, 'n_estimators': 100} | 0.026372794251142438 | 0.9449651272738459 | 0.0408116991409925 |

Yeast_1: *S. cerevisiae,* Yeast_2: *L. thermotolerans*, Yeast_3: *T. delbrueckii*, Yeast_4: *W. anomalus*

**
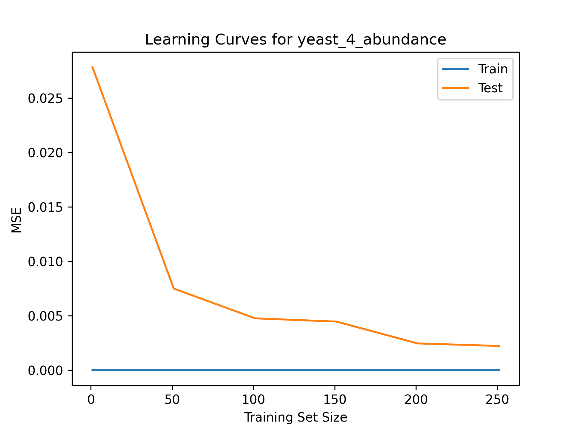

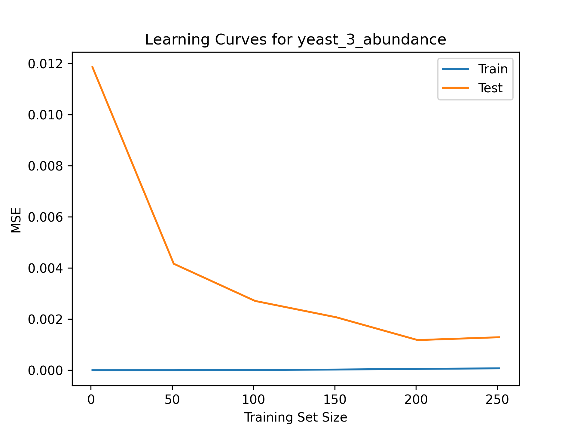

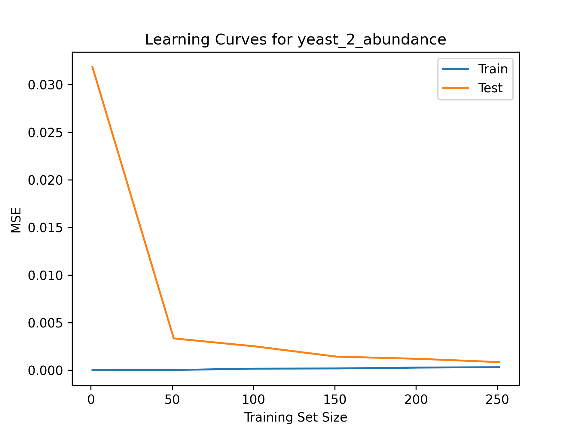

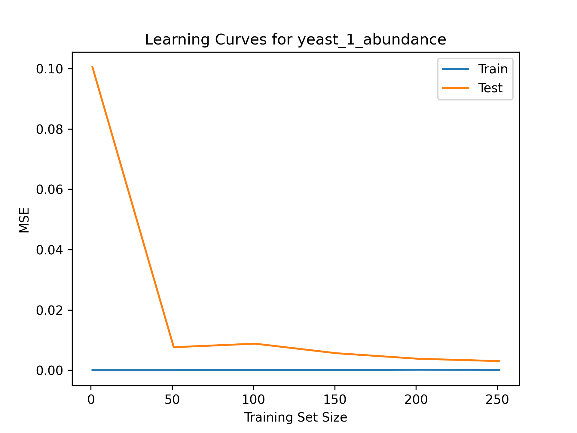

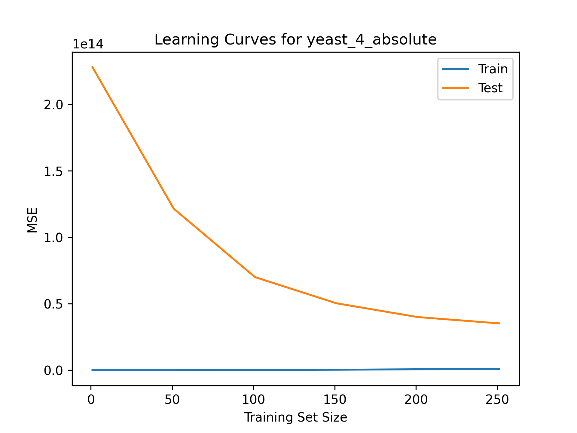

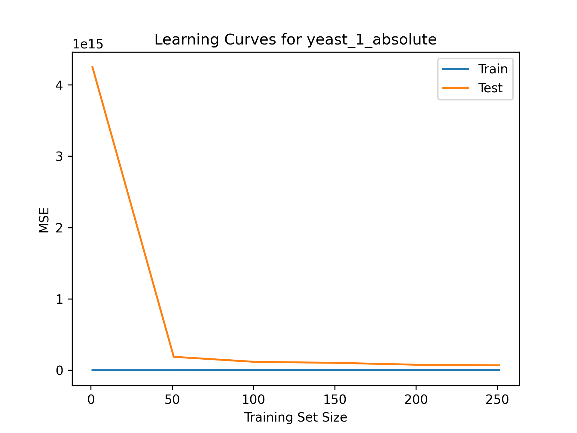

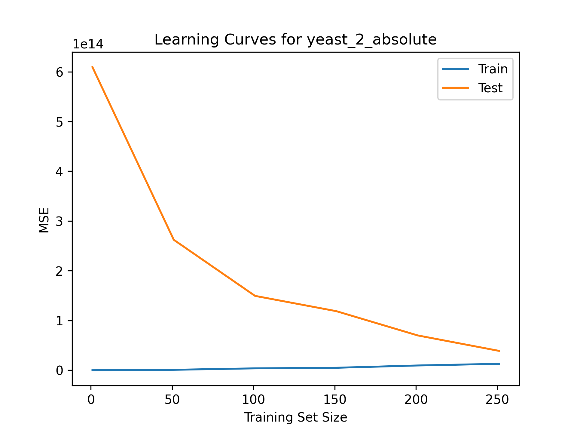

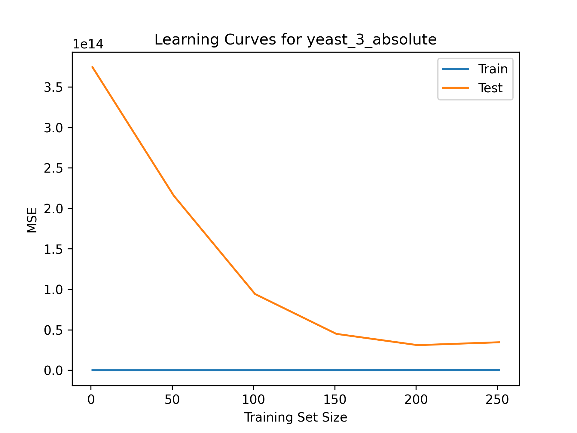
Supp. Fig. 8**. Comparative learning curves of Gradient Boosting Regressor for each target variable of the model trained on the community-data subset. Each subfigure (A-I) illustrates the learning curve for a separate target variable, with the x-axis representing the number of training examples and the y-axis denoting the model's performance metric (mean squared error). The blue line plots the performance on the training set, while the orange line denotes the performance on the validation set. The proximity of these two lines and their convergence pattern provide an indication of the model's learning progress and potential for improvement with additional data. Subfigures: (A) yeast_1_absolute, (B) yeast_2_absolute, (C) yeast_3_absolute, (D) yeast_4_absolute, (E) yeast_1_abundance, (F) yeast_2_abundance, (G) yeast_3_abundance, and (H) yeast_4_abundance.

**C**

**B**

**A**

**D**

**E**

**F**

**G**

**H**


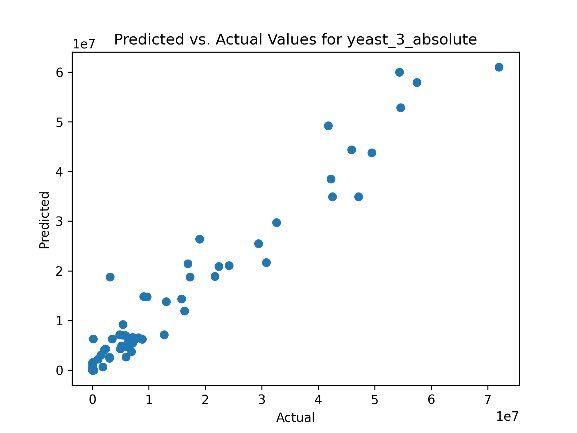

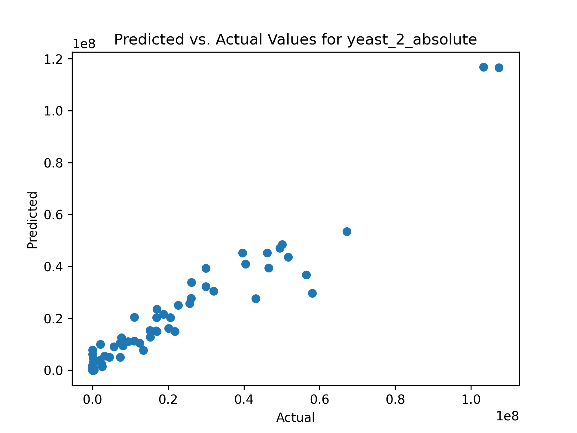

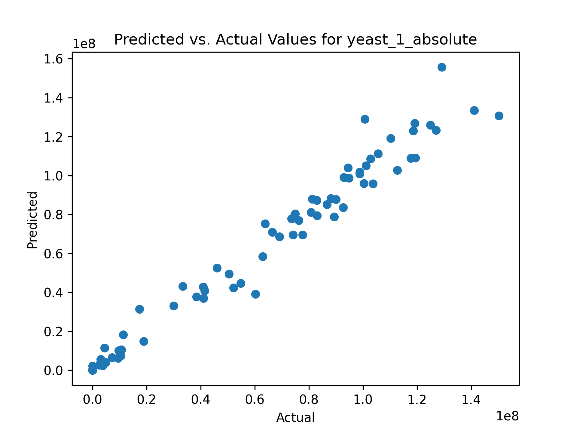

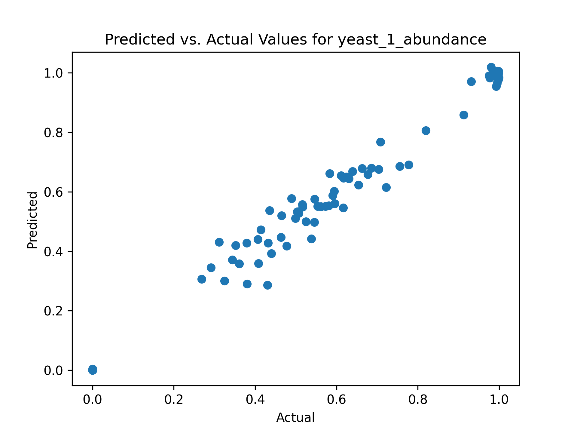

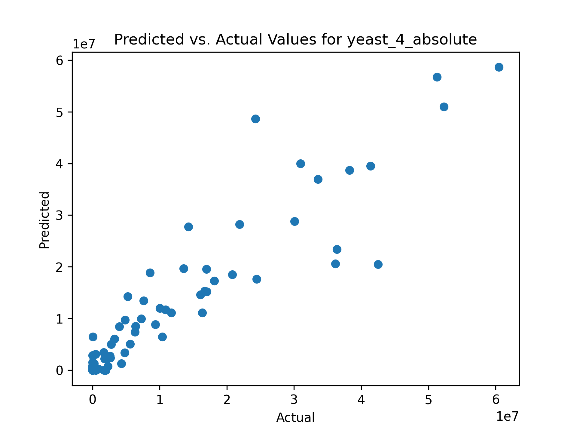

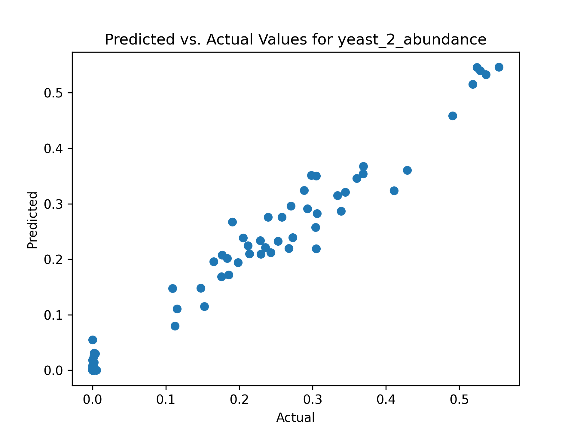

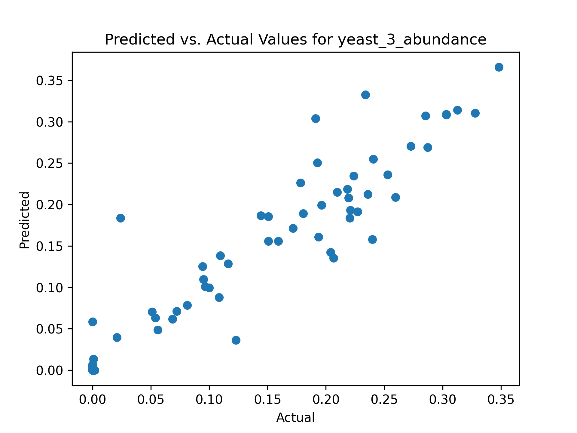

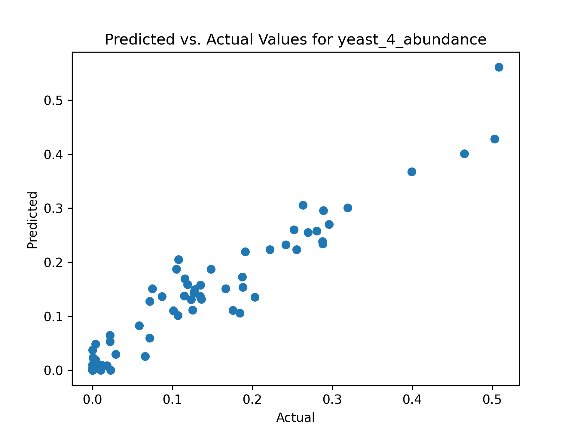


**A**

**H**

**G**

**F**

**E**

**D**

**C**

**B**

**Supp. Fig. 9.** Predicted versus actual value plots for each target variable in the Gradient Boosting Regressor model trained on the community-data subset. Each subfigure (A-I) shows a scatter plot where each point represents a particular sample. The x-axis denotes the actual values, and the y-axis denotes the predicted values for each target. A perfect model would result in all points aligning along the diagonal line, which represents a one-to-one correspondence between predicted and actual values. Deviations from this line indicate prediction errors. Subfigures: (A) yeast_1_absolute, (B) yeast_2_absolute, (C) yeast_3_absolute, (D) yeast_4_absolute, (E) yeast_1_abundance, (F) yeast_2_abundance, (G) yeast_3_abundance, and (H) yeast_4_abundance.


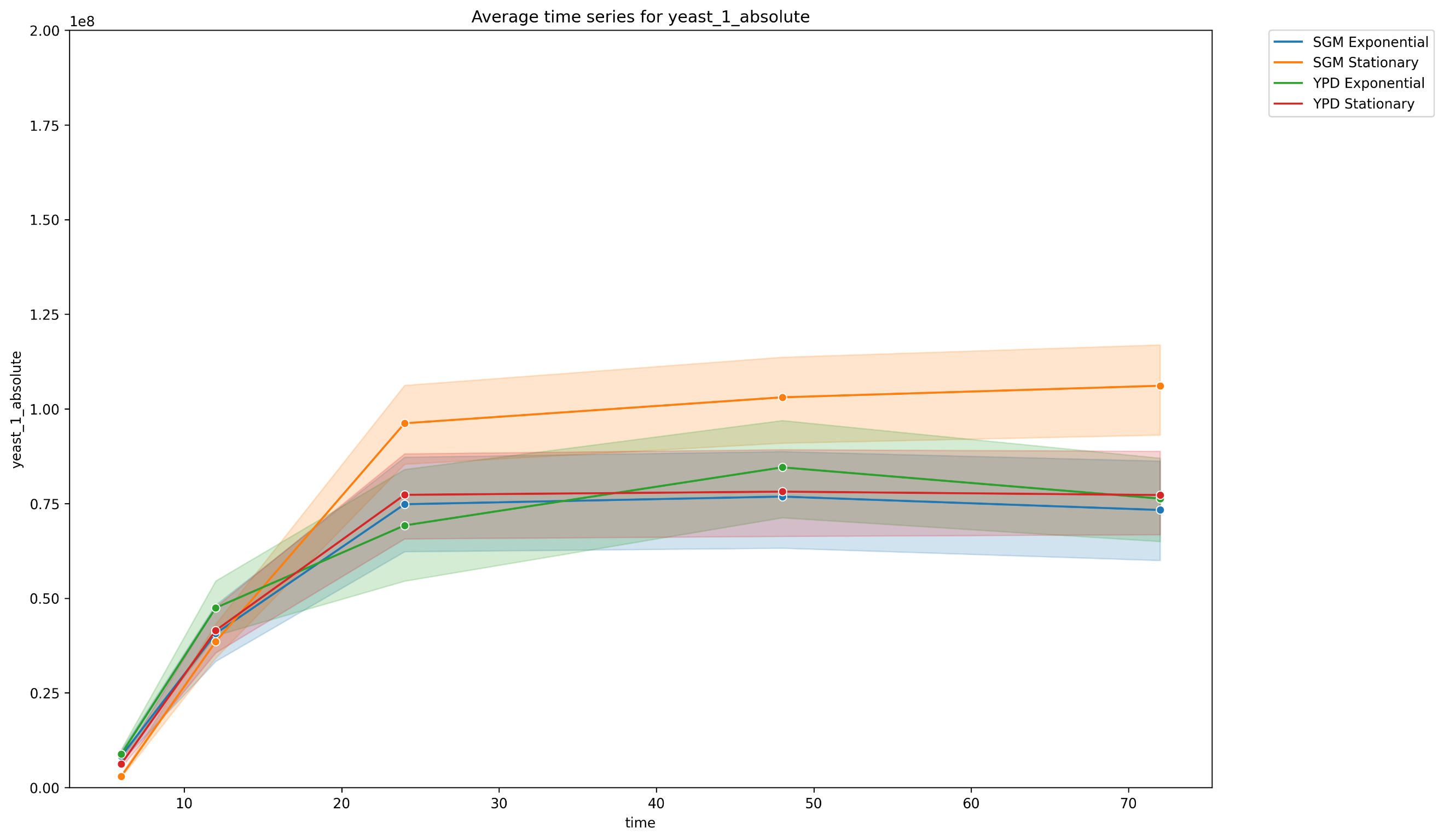

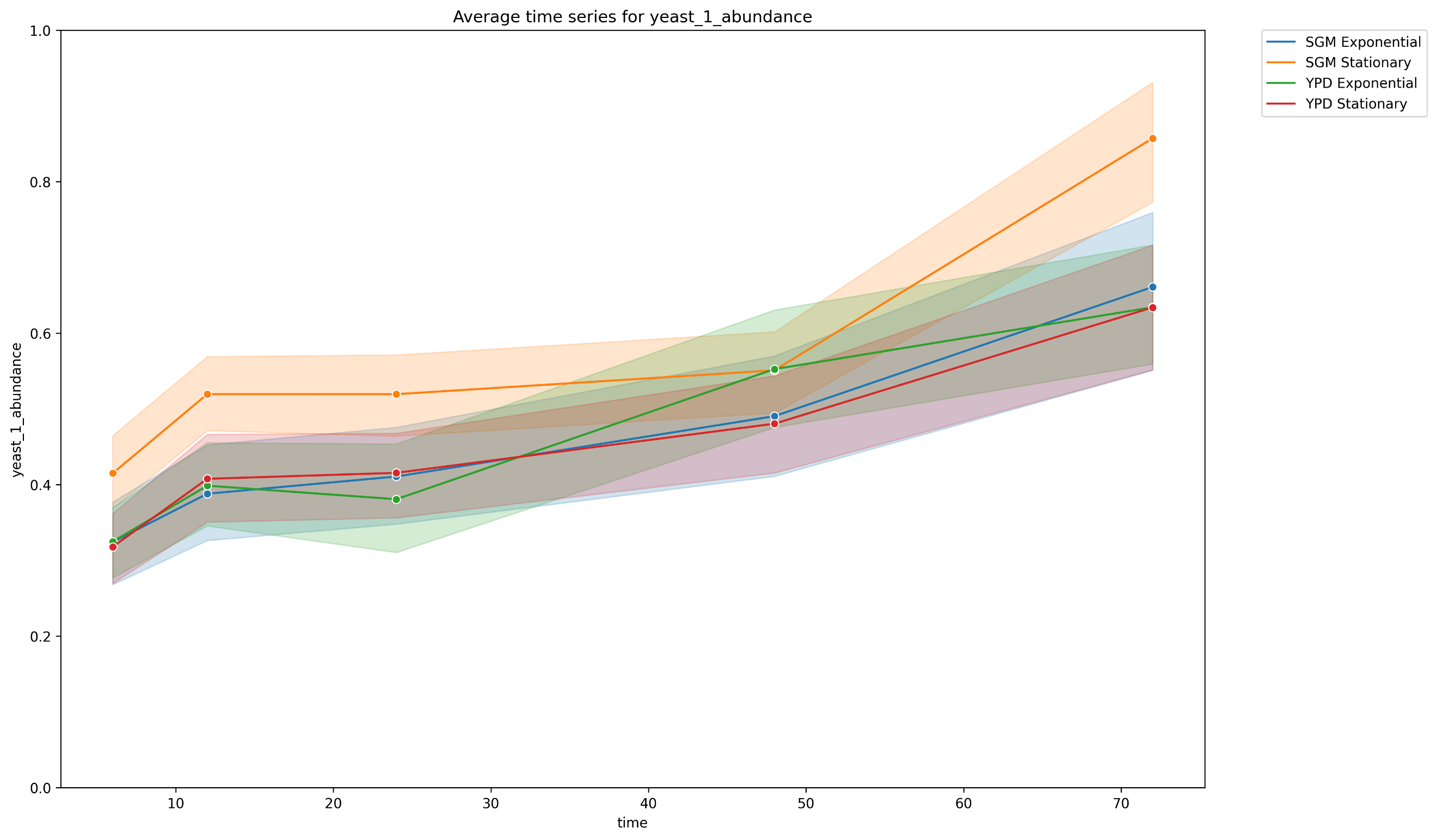


**B**

**A**


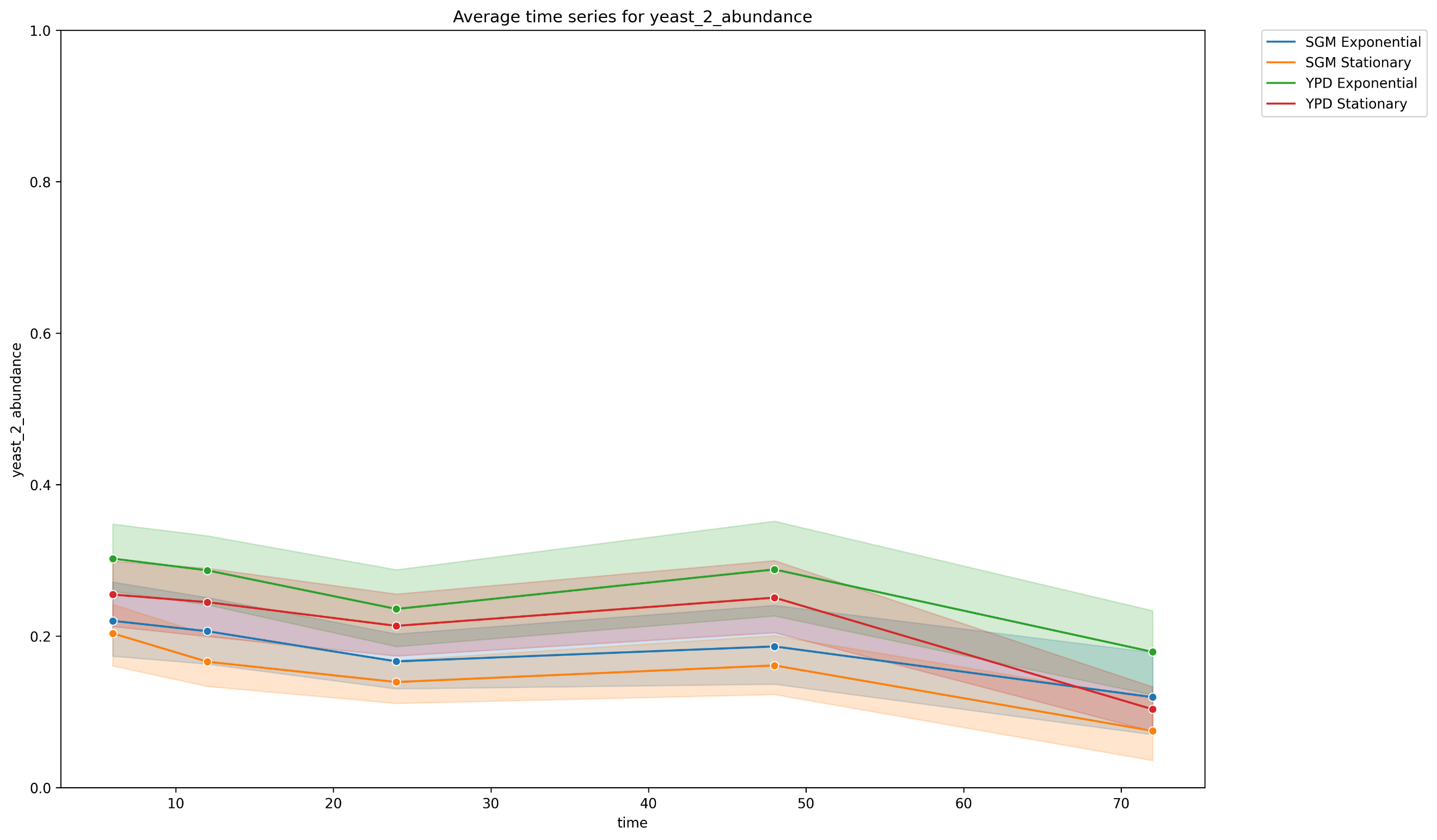

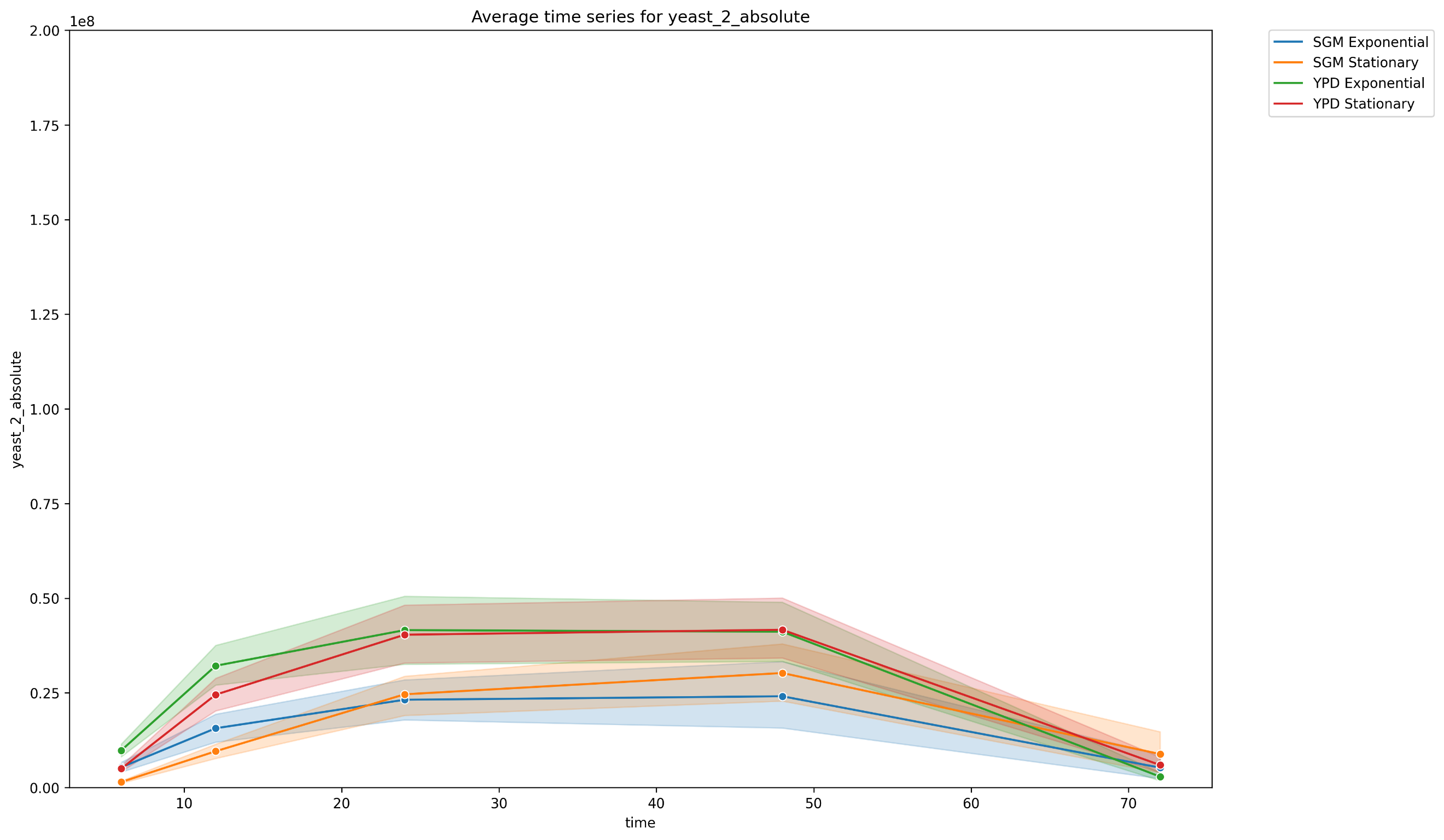


**D**

**C**


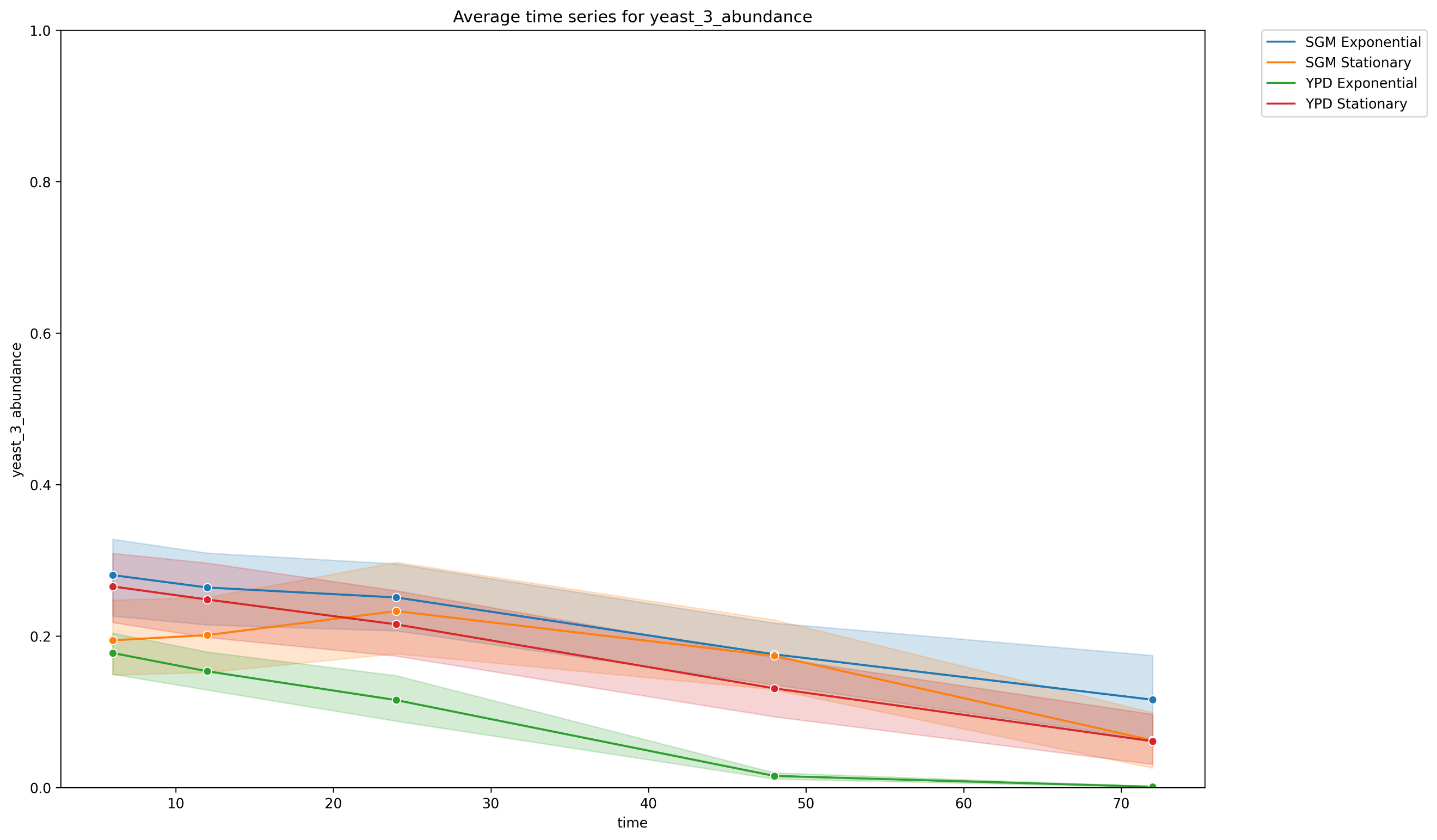

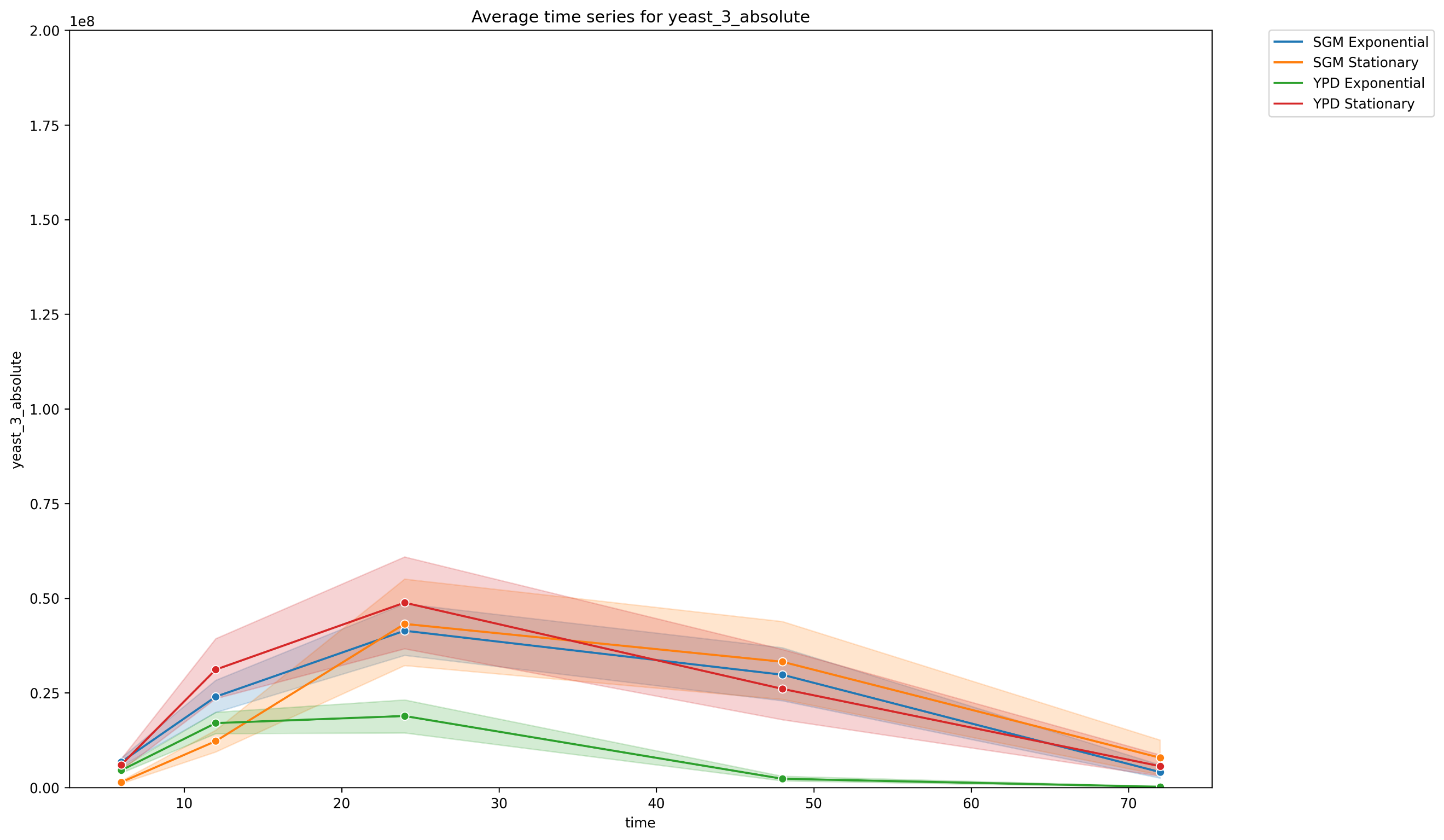


**F**

**E**


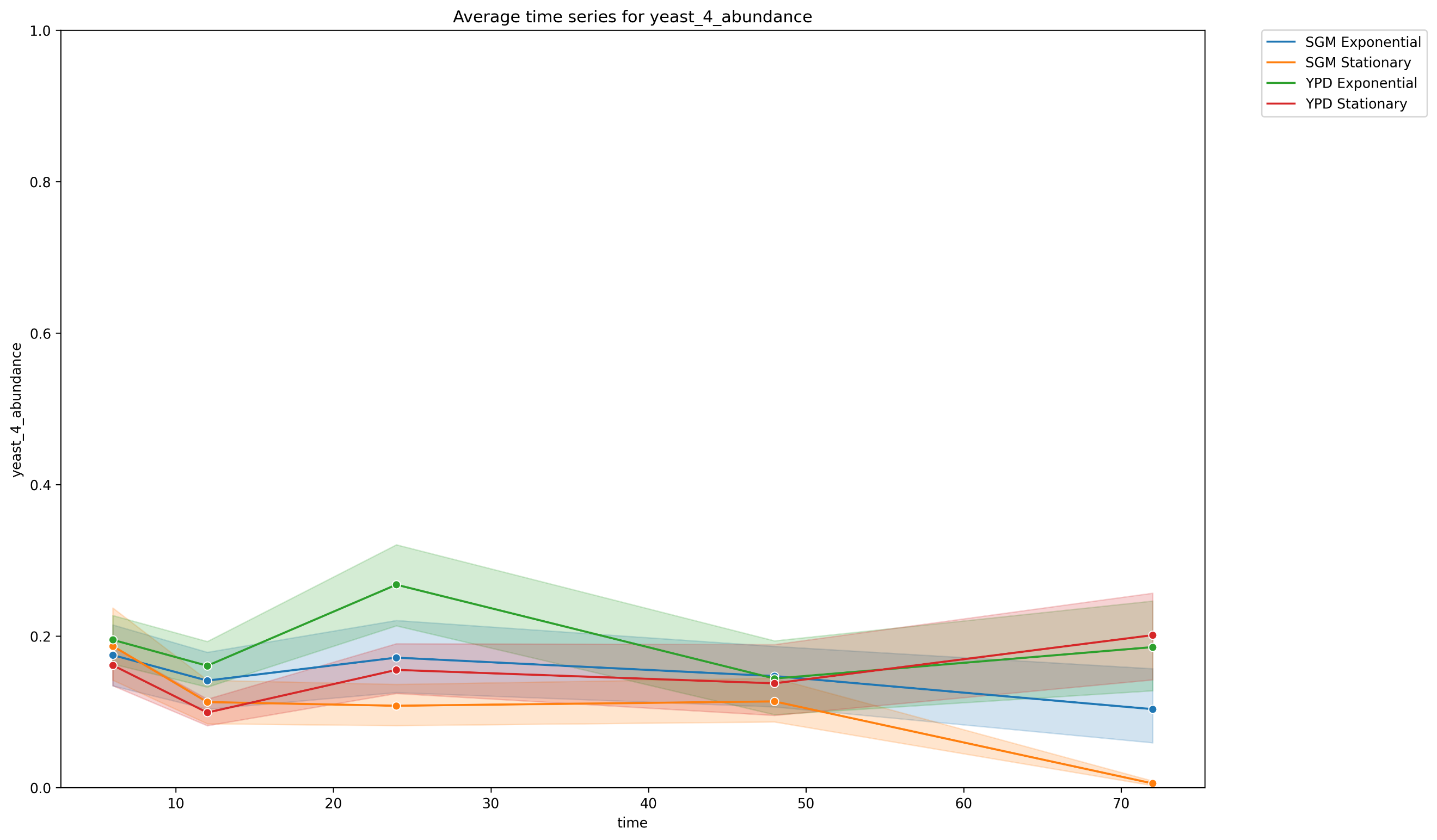

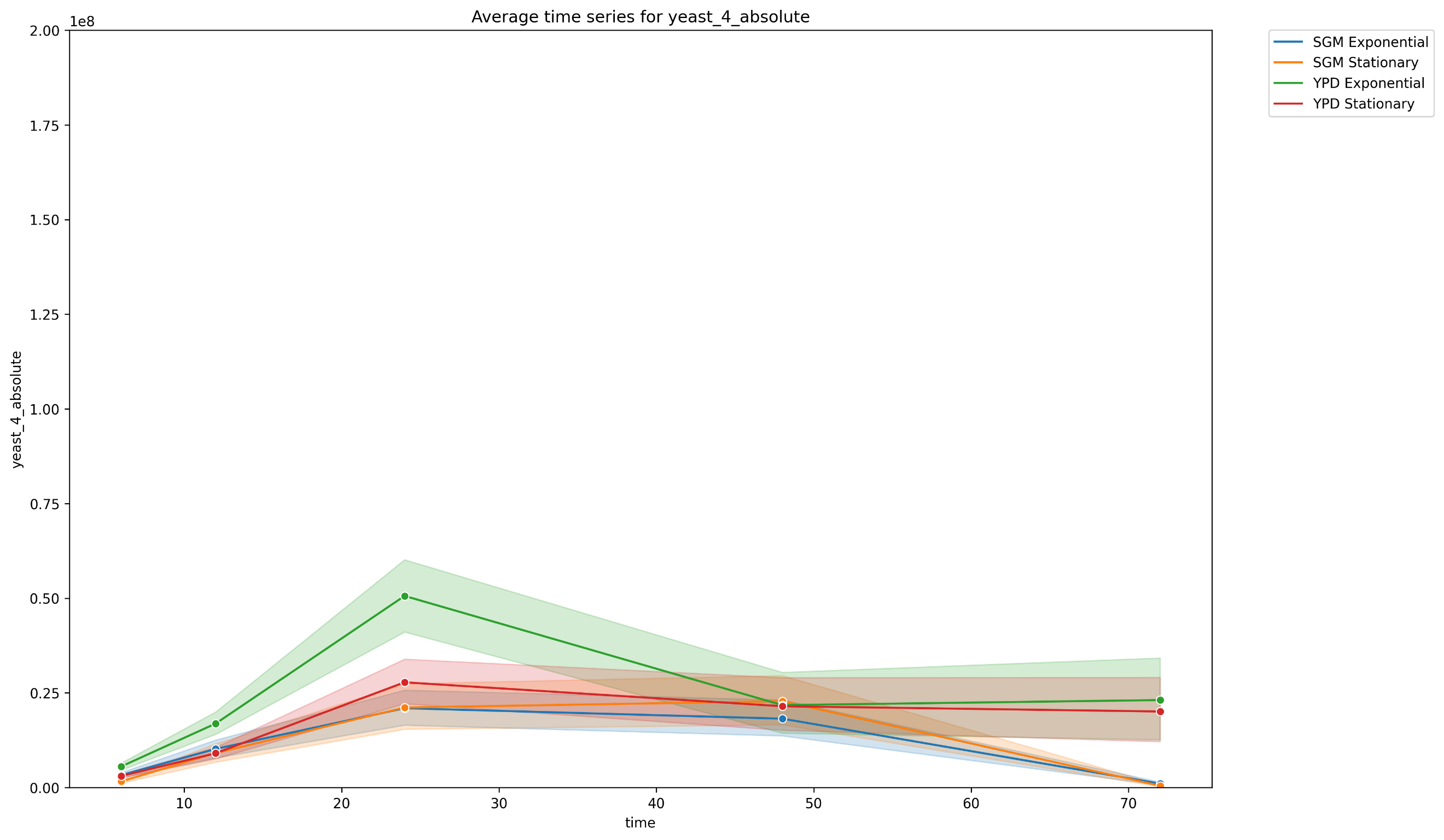


**H**

**G**

**Supp. Fig. 10.** Time-series absolute and relative abundance of each species in all mixed culture conditions, averaged across different pre-culturing conditions. Two growth media, namely Synthetic Grape Must (SGM) and Yeast Peptone Dextrose (YPD) were combined with two different growth phases, namely exponential and stationary growth phase. Red line: YPD + Stationary, Green line: YPD + Exponential, Orange line: SGM + Stationary, Blue line: SGM + Exponential. Subfigures: (A) yeast_1_absolute, (B) yeast_1_abundance, (C) yeast_2_absolute, (D) yeast_2_abundance (E) yeast_3_absolute, (F) yeast_3_abundance, (G) yeast_4_absolute, and (H) yeast_4_abundance.

**Supp. Fig. 11.** Feature importance values for gradient boosted regression models trained on subsets of the dataset, including either 2 (A, C, E, G) or more than 2 (B, D, F, H) species in the multispecies culture. The model targets were all absolute abundance. Feature values are reported for each species within the community, namely *S. cerevisiae* (A, B), *L. thermotolerans* (C, D), *T. delbrueckii* (E, F), and *W. anomalus* (G, H).
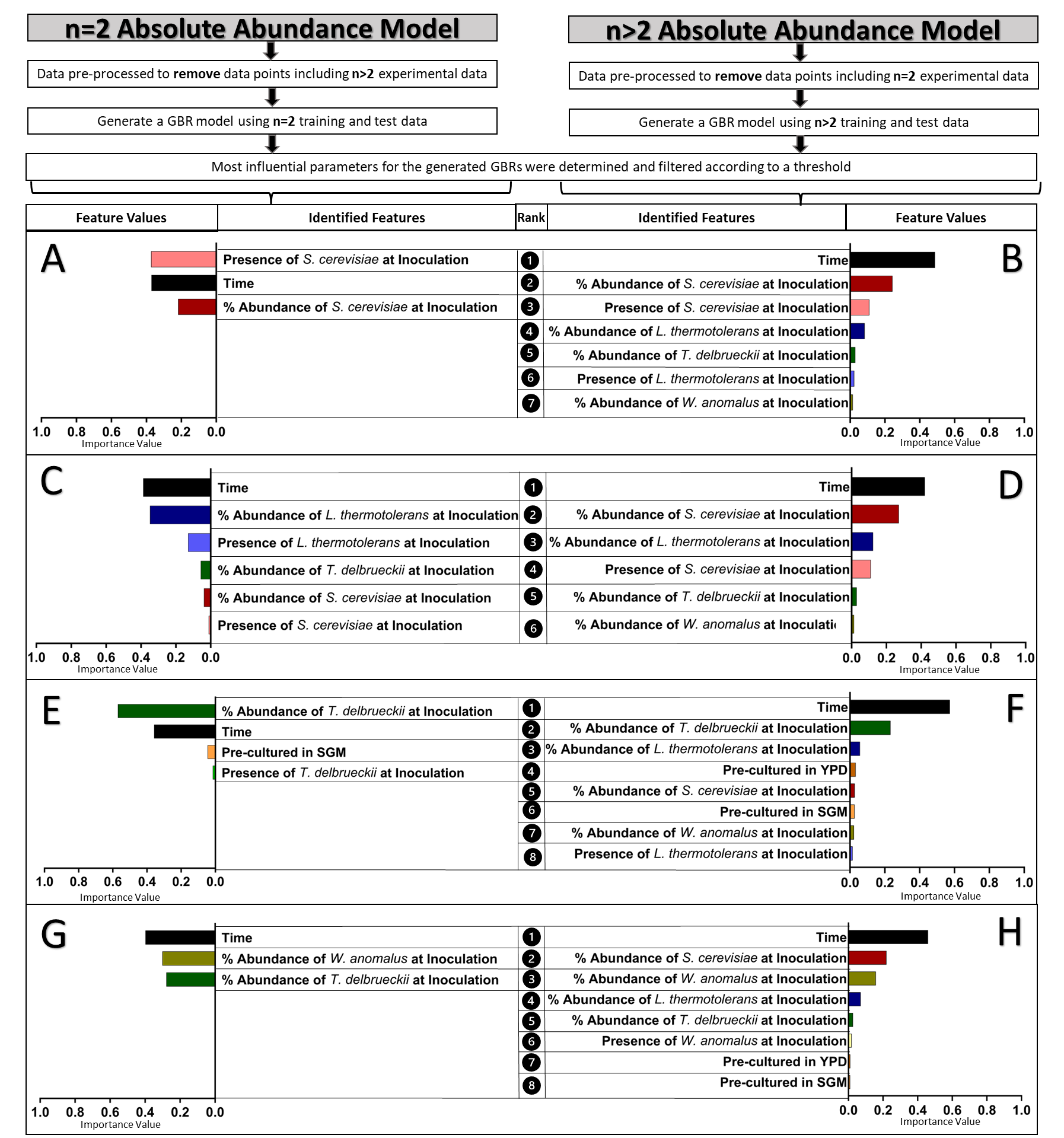


**Supp. Table 3**. Model metrics for model generated with Bagheri et al. 2020 dataset.

| Target | Best Parameters | MAE | RMSE | CCC |
| --- | --- | --- | --- | --- |
| yeast_1_abundance | {'learning_rate': 0.1, 'max_depth': 5, 'n_estimators': 100} | 0.0035464006851047224 | 0.004773132903338527 | 0.9636102501952274 |
| yeast_2_abundance | {'learning_rate': 0.2, 'max_depth': 3, 'n_estimators': 50} | 0.006299126079221048 | 0.007869434047975365 | 0.9953594549462313 |
| yeast_3_abundance | {'learning_rate': 0.2, 'max_depth': 4, 'n_estimators': 100} | 0.005976497894344197 | 0.007031966041696229 | 0.9987592660754768 |
| yeast_4_abundance | {'learning_rate': 0.2, 'max_depth': 3, 'n_estimators': 100} | 0.004710362856227211 | 0.00572201686288911 | 0.9936849588857679 |
| yeast_5_abundance | {'learning_rate': 0.2, 'max_depth': 3, 'n_estimators': 200} | 0.005177736895197854 | 0.006197621696948248 | 0.9987290685656992 |
| yeast_6_abundance | {'learning_rate': 0.2, 'max_depth': 4, 'n_estimators': 50} | 0.005656388843259581 | 0.00646532317026283 | 0.9982122382053424 |
| yeast_7_abundance | {'learning_rate': 0.1, 'max_depth': 4, 'n_estimators': 100} | 0.0037752246902610217 | 0.004574381091552821 | 0.9987665243187515 |
| yeast_8_abundance | {'learning_rate': 0.1, 'max_depth': 3, 'n_estimators': 200} | 0.0066605740529058054 | 0.008235107887317322 | 0.9994235722373396 |
| yeast_10_abundance | {'learning_rate': 0.2, 'max_depth': 4, 'n_estimators': 100} | 0.0038461556923497313 | 0.004993418630180895 | 0.9991628264418587 |


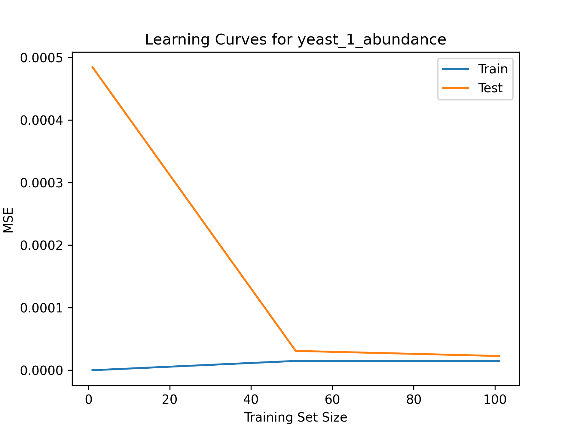

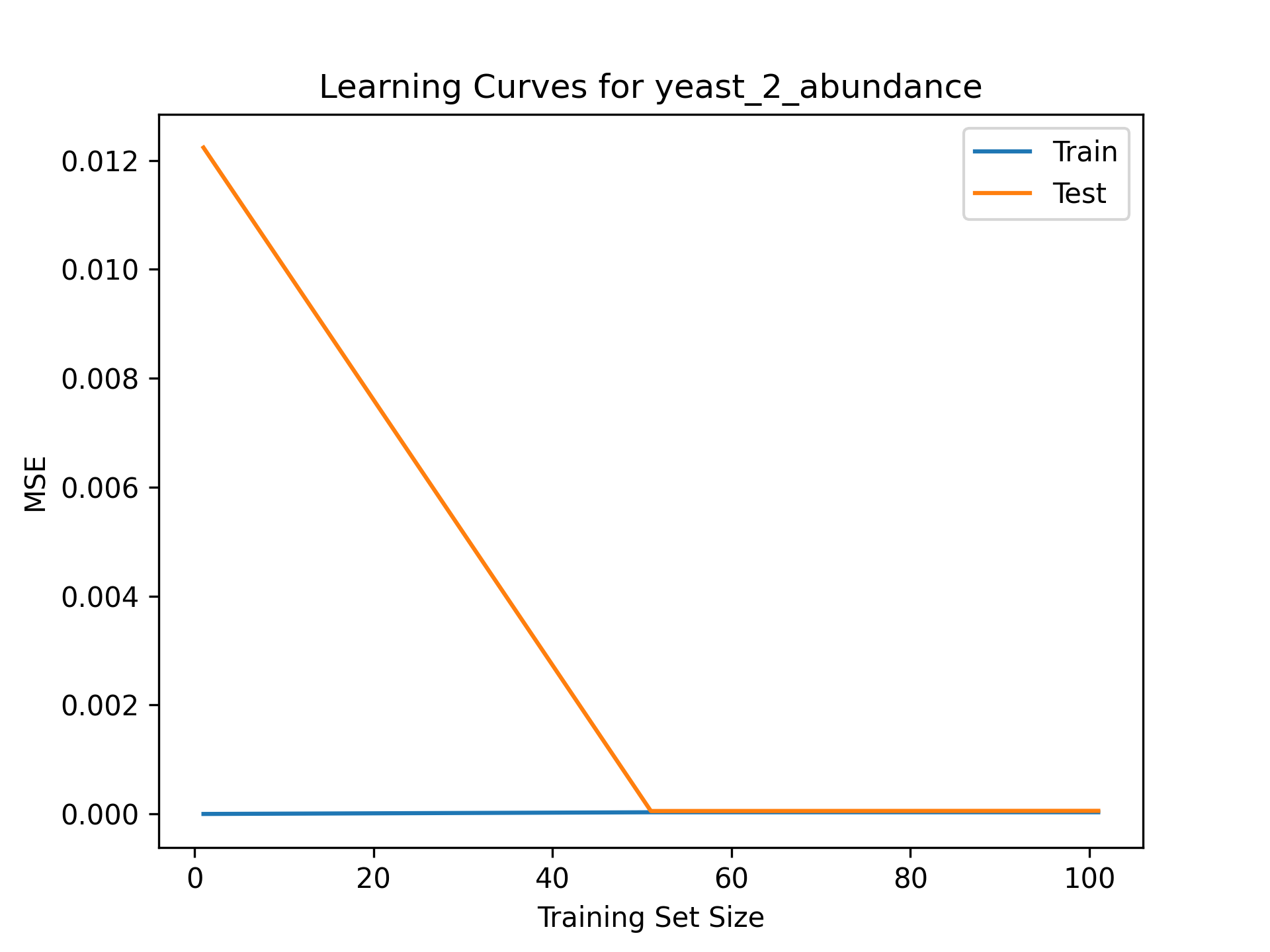

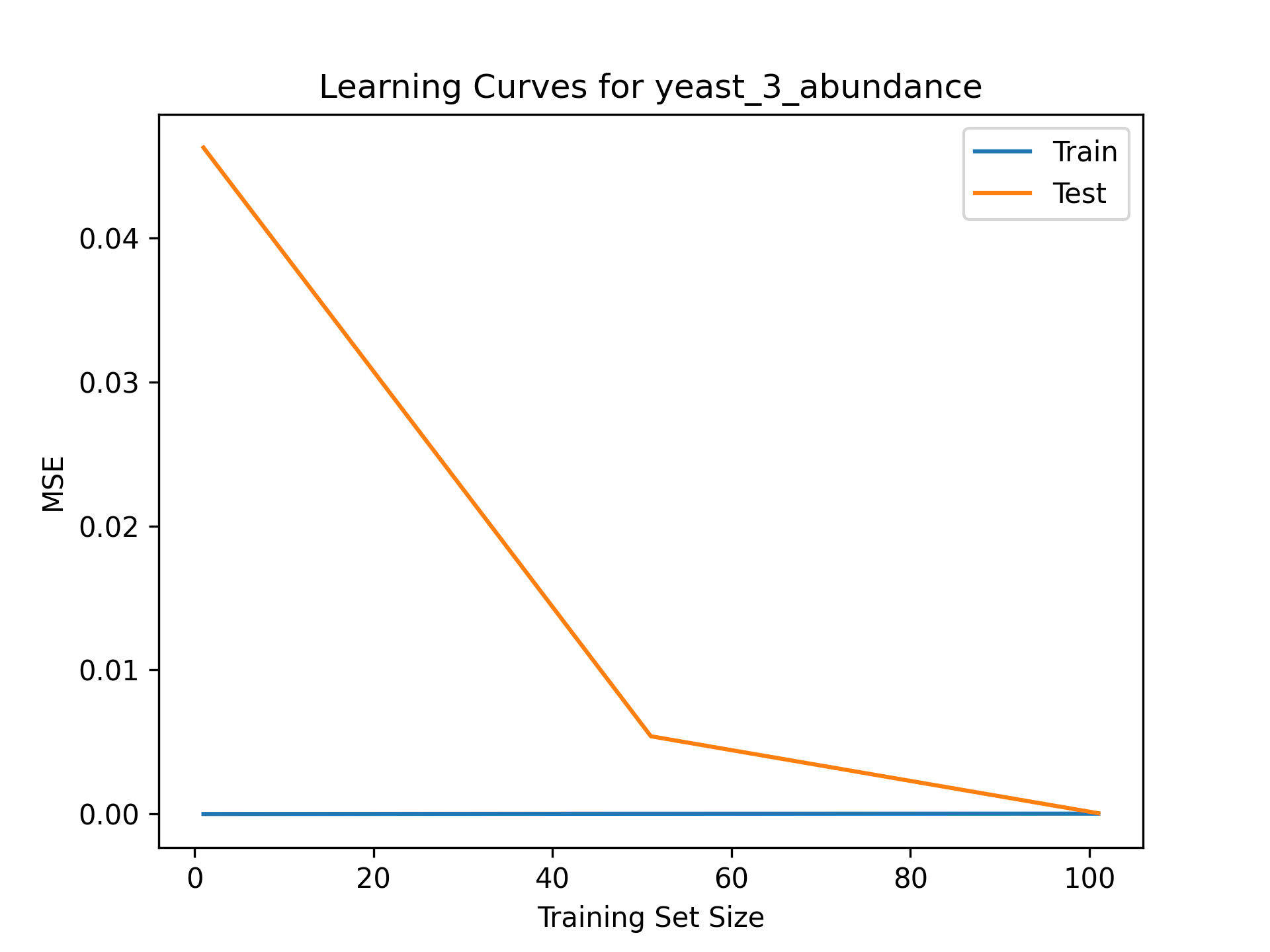

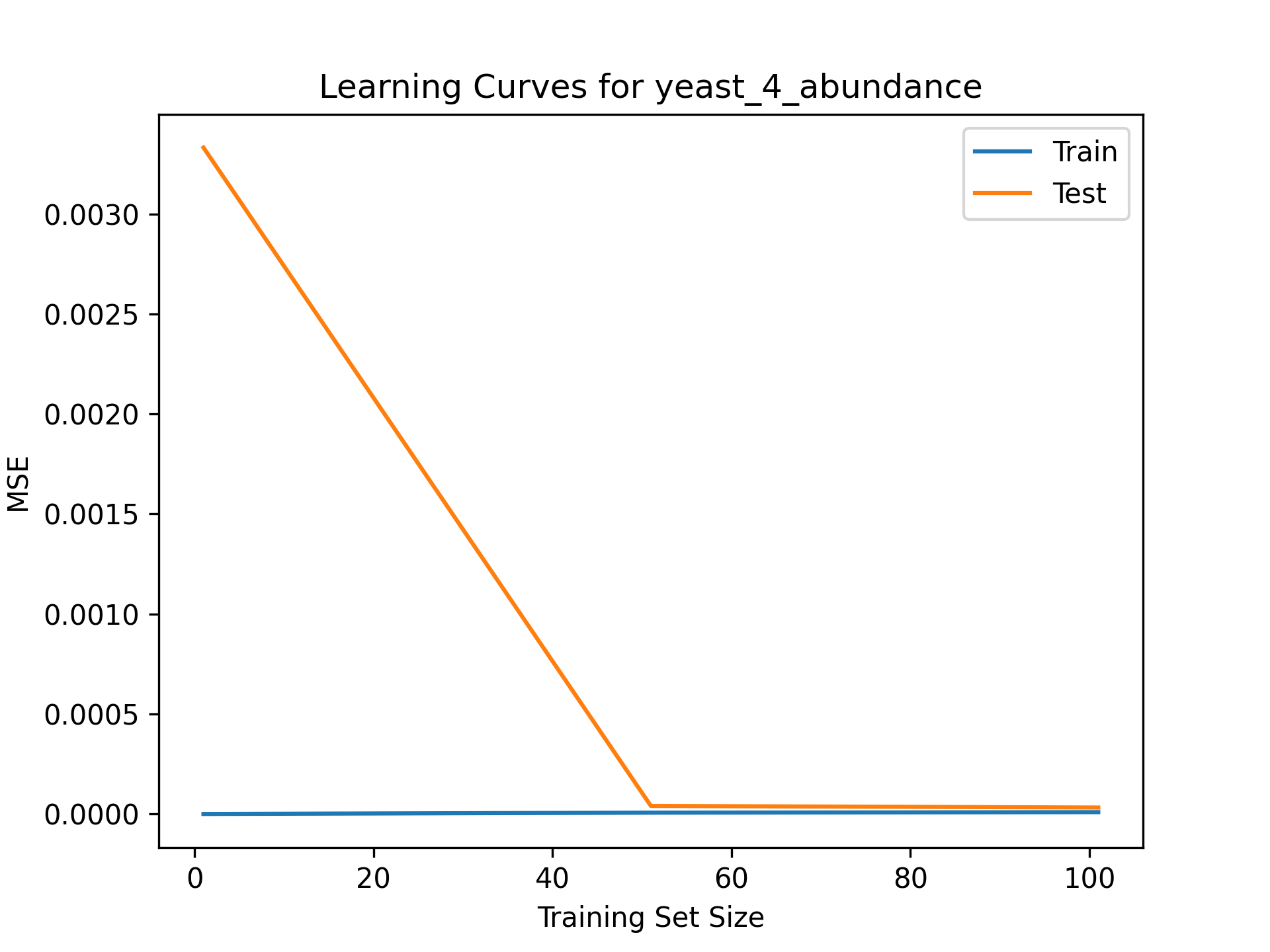

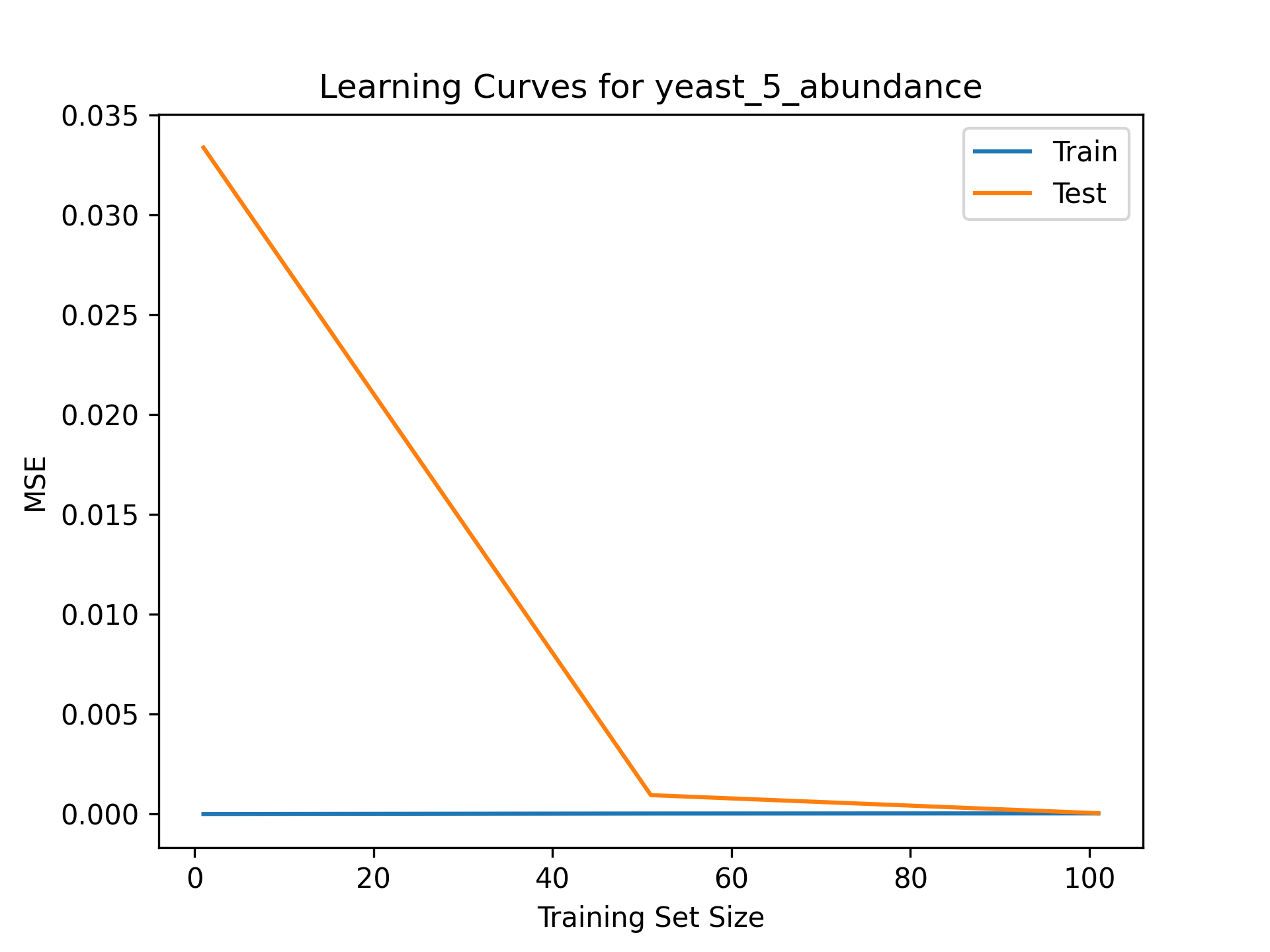

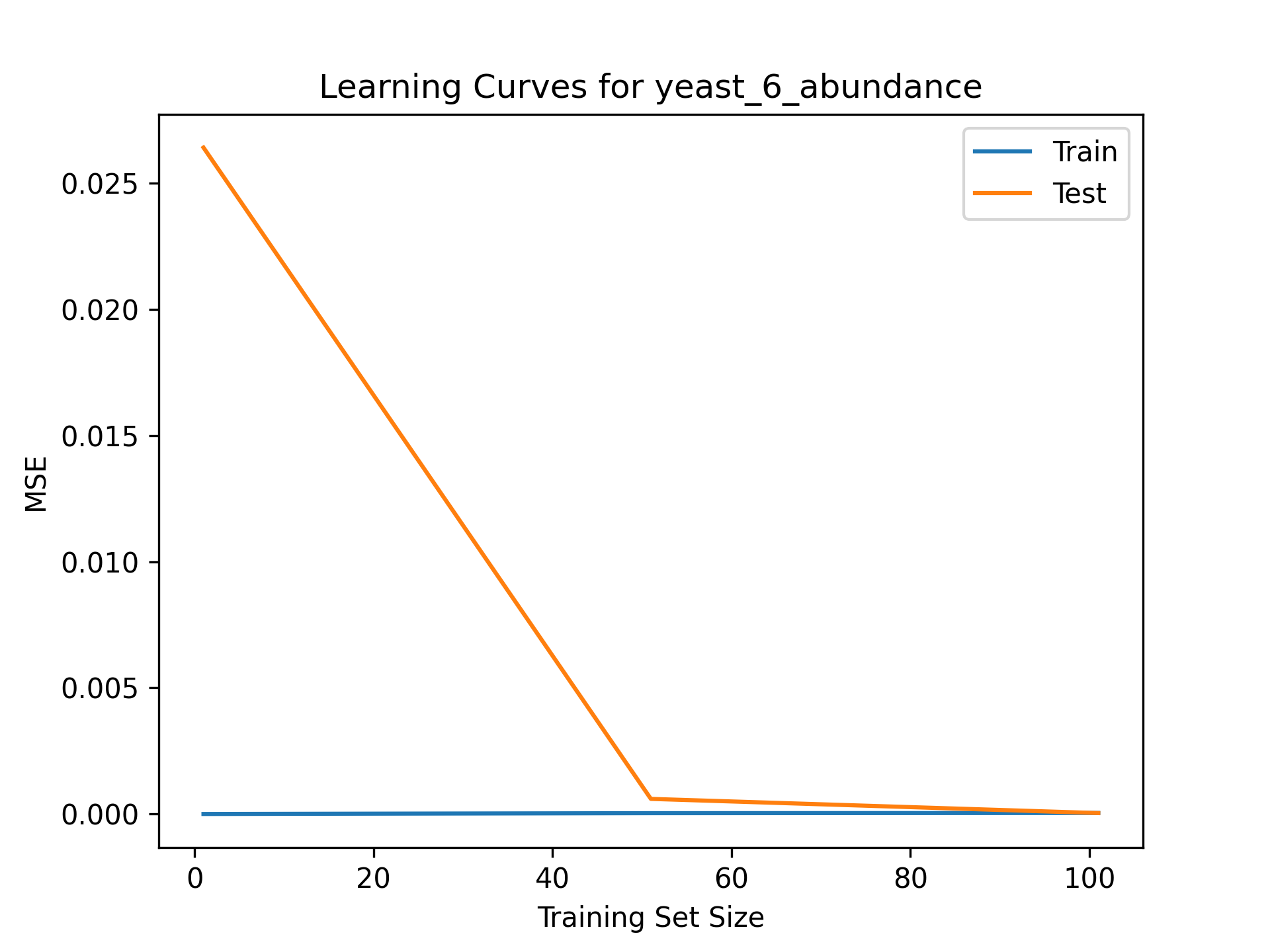

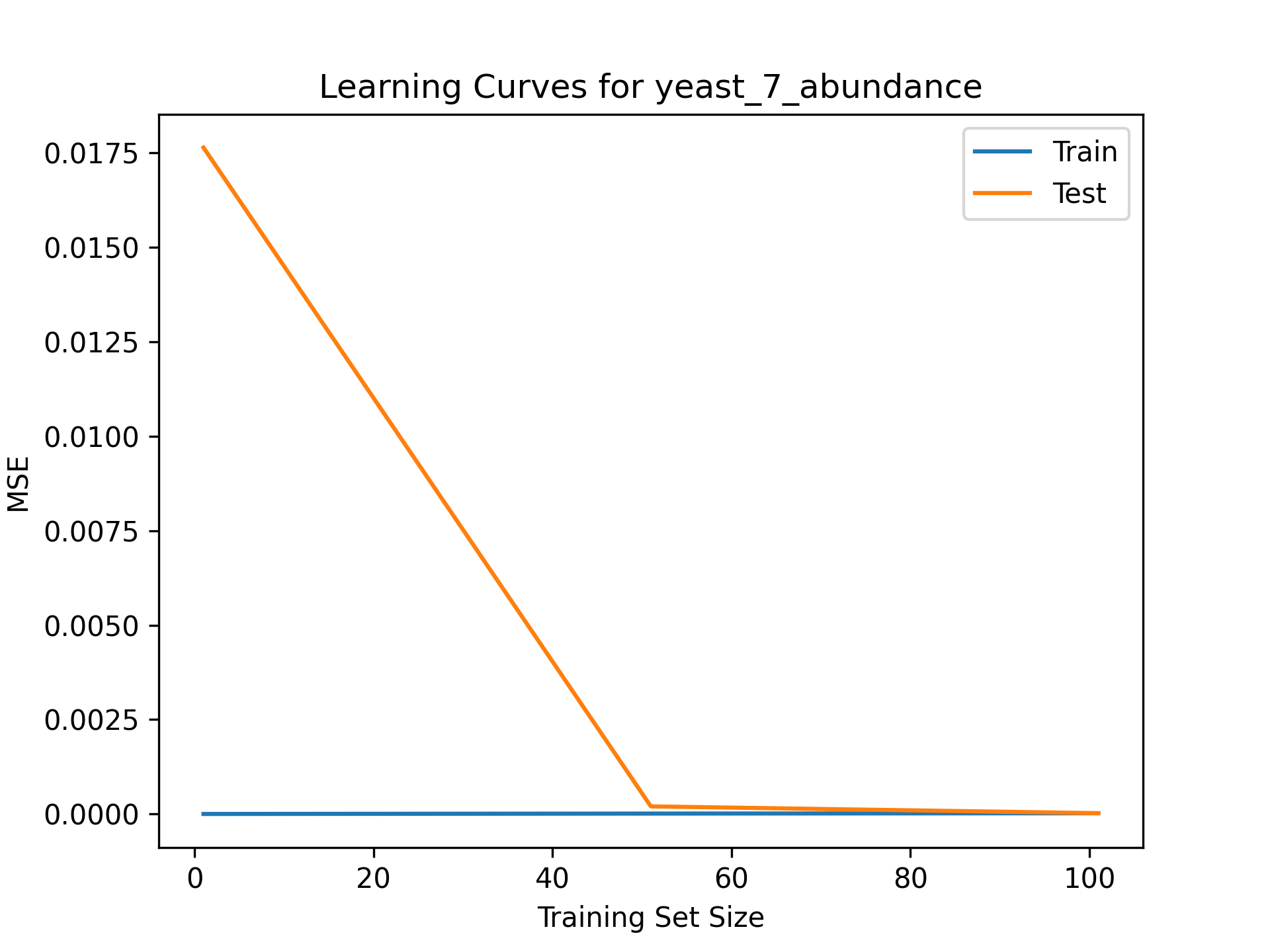

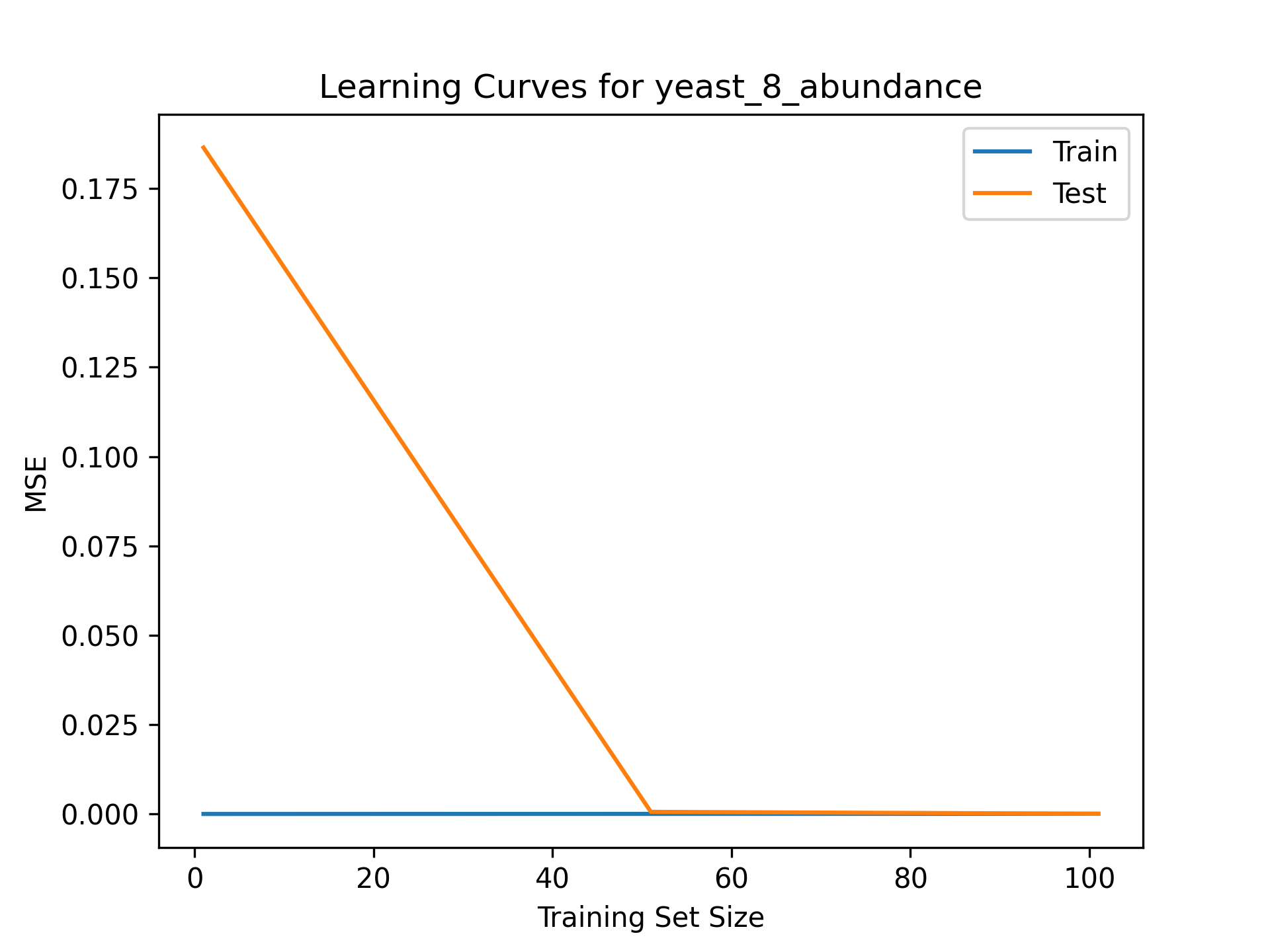

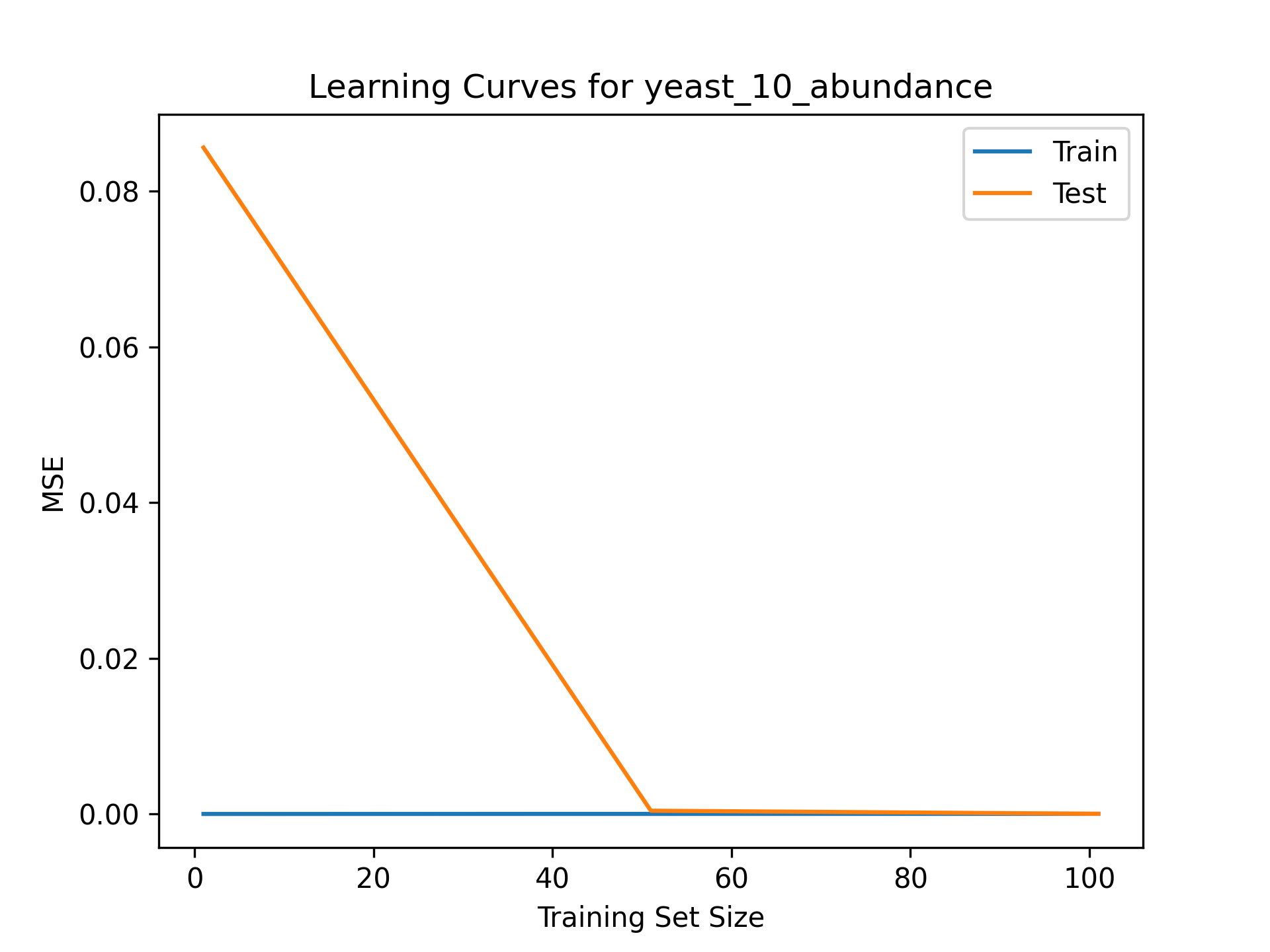


**A**

**B**

**C**

**D**

**E**

**F**

**G**

**H**

**J**

**Supp. Fig. 12.** Comparative learning curves of Gradient Boosting Regressor for each target variable for the model trained on the Bagheri et al dataset supplemented with synthesized data. Each subfigure (A-I) illustrates the learning curve for a separate target variable, with the x-axis representing the number of training examples and the y-axis denoting the model's performance metric (mean squared error). The blue line plots the performance on the training set, while the orange line denotes the performance on the validation set. The proximity of these two lines and their convergence pattern provide an indication of the model's learning progress and potential for improvement with additional data. Subfigures: (A) yeast_1_abundance, (B) yeast_2_abundance, (C) yeast_3_abundance, (D) yeast_4_abundance, (E) yeast_5_abundance, (F) yeast_6_abundance, (G) yeast_7_abundance, (H) yeast_8_abundance, (I) yeast_9_abundance and (J) yeast_10_abundance.


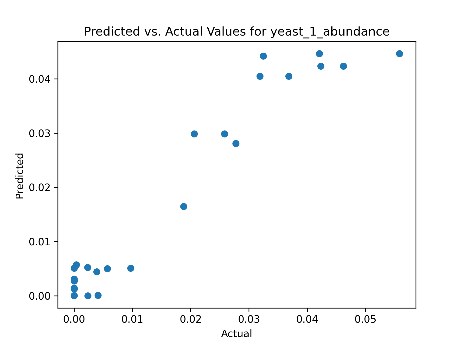

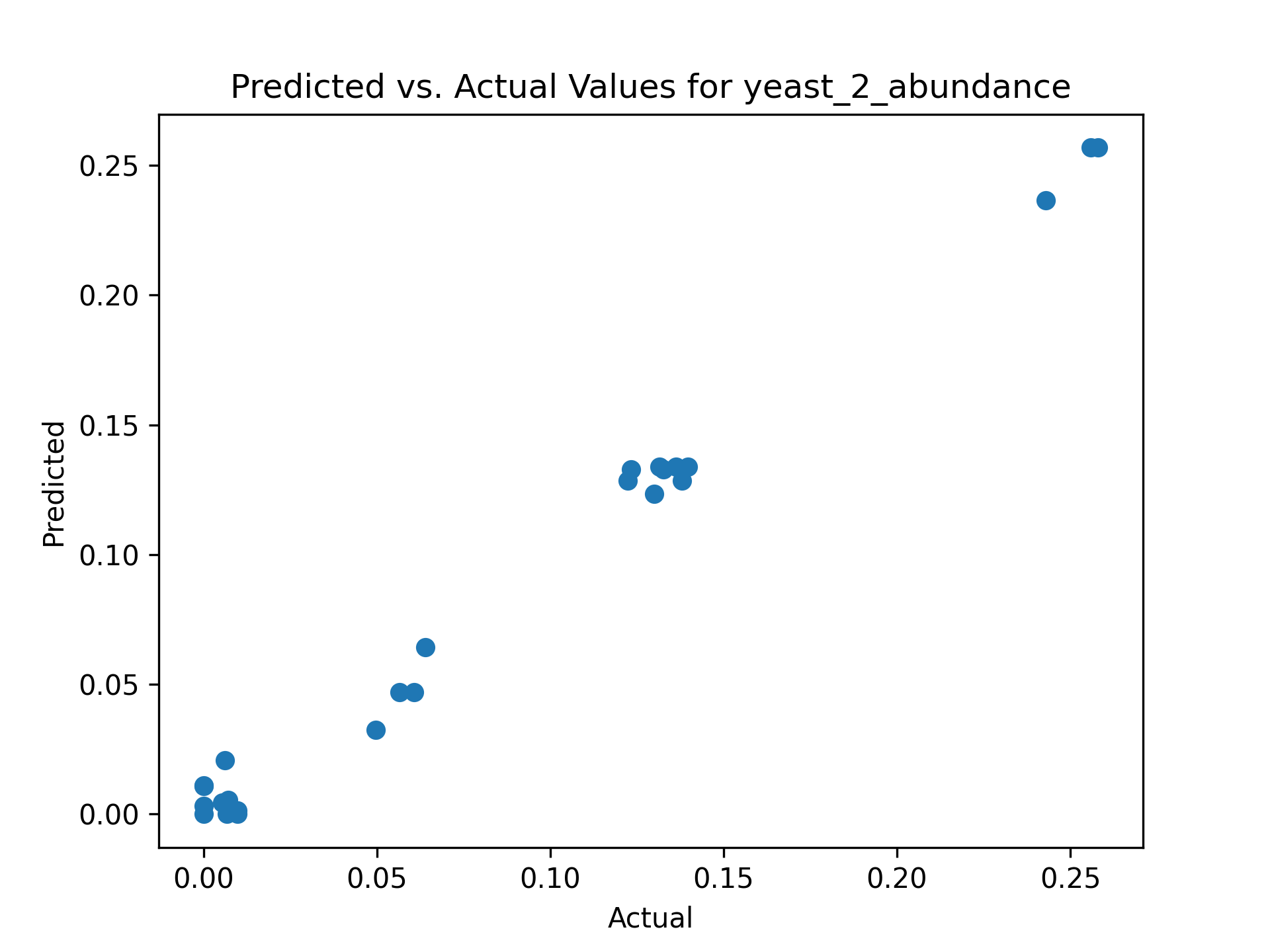

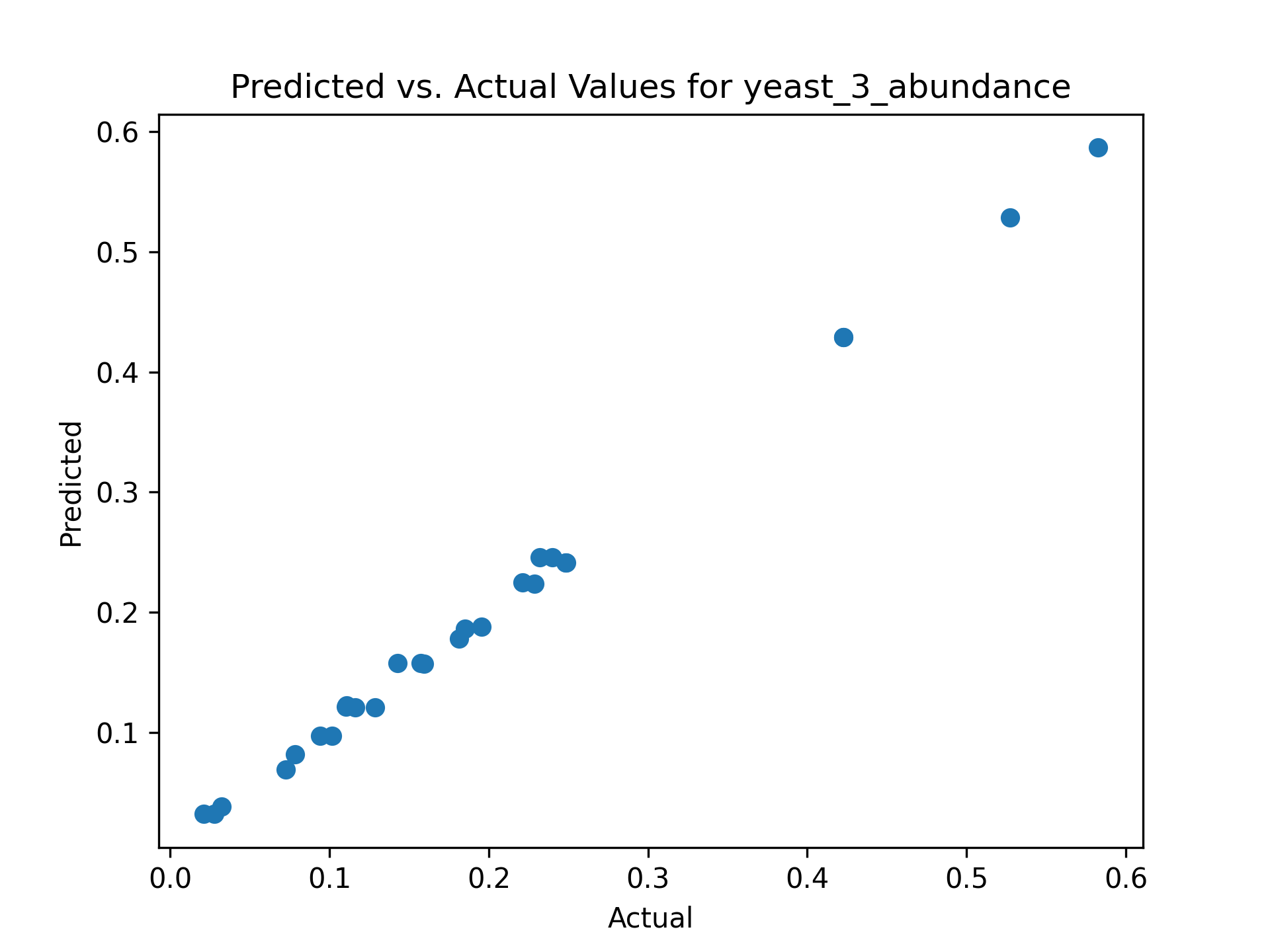

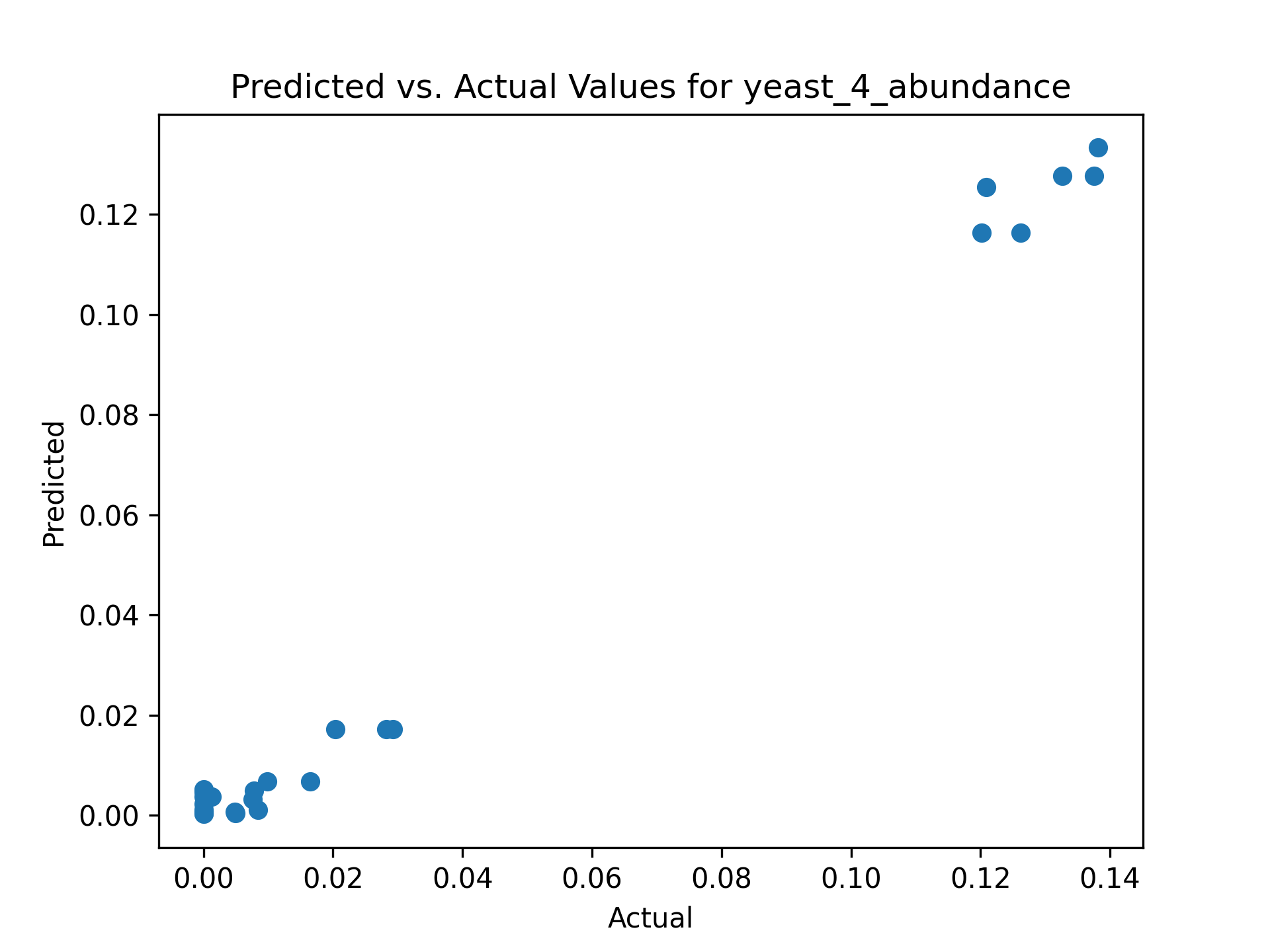

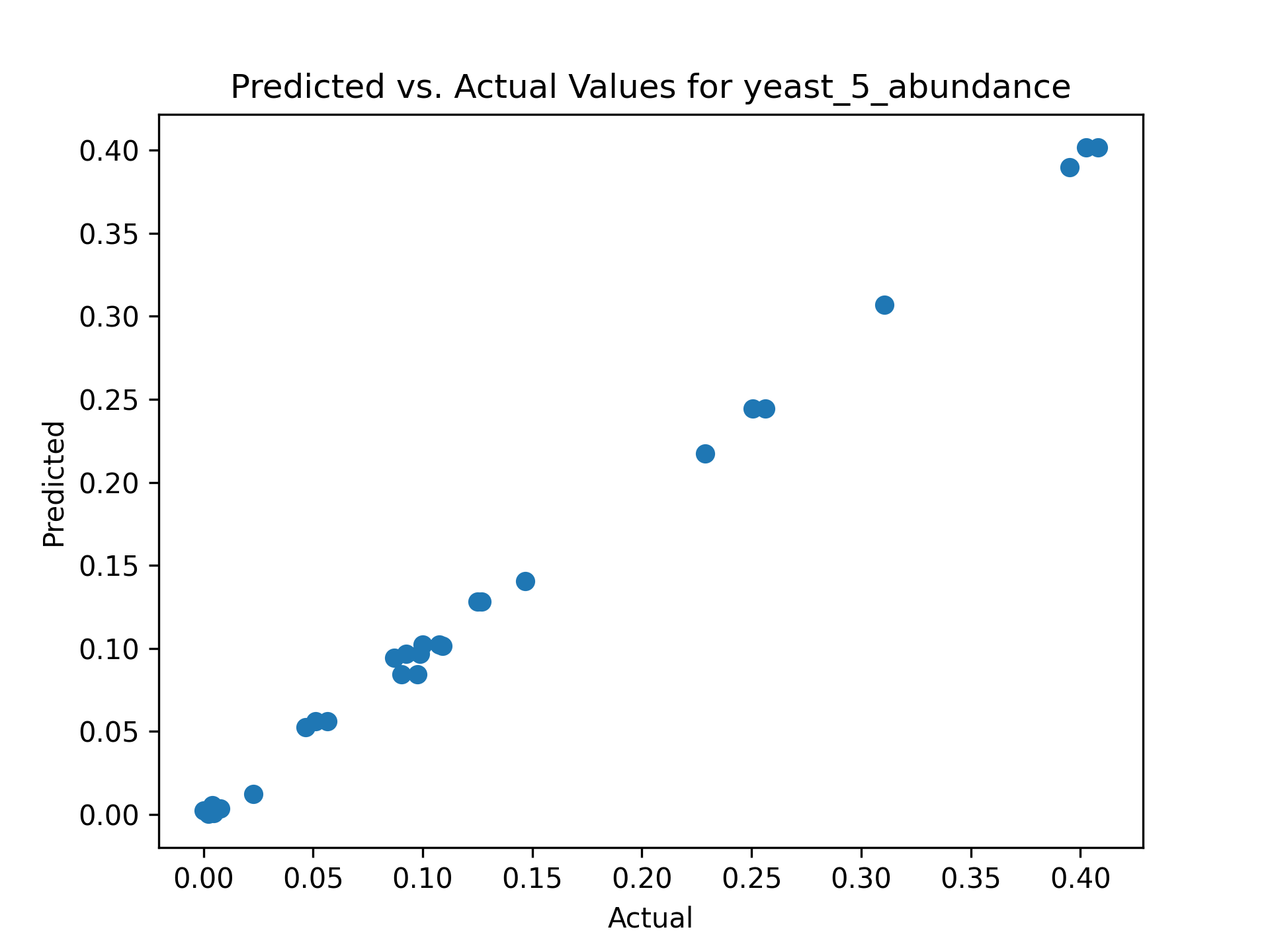

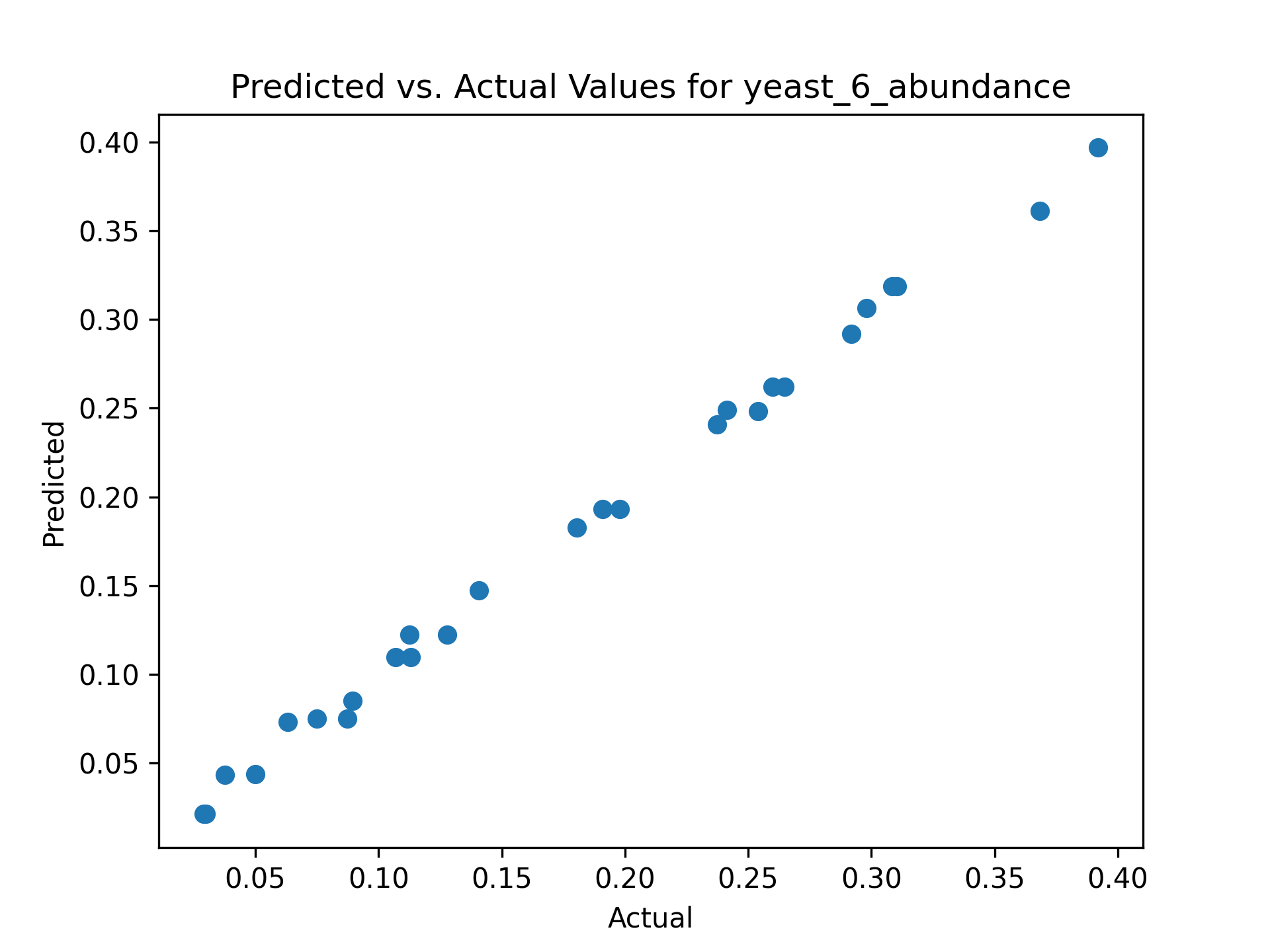

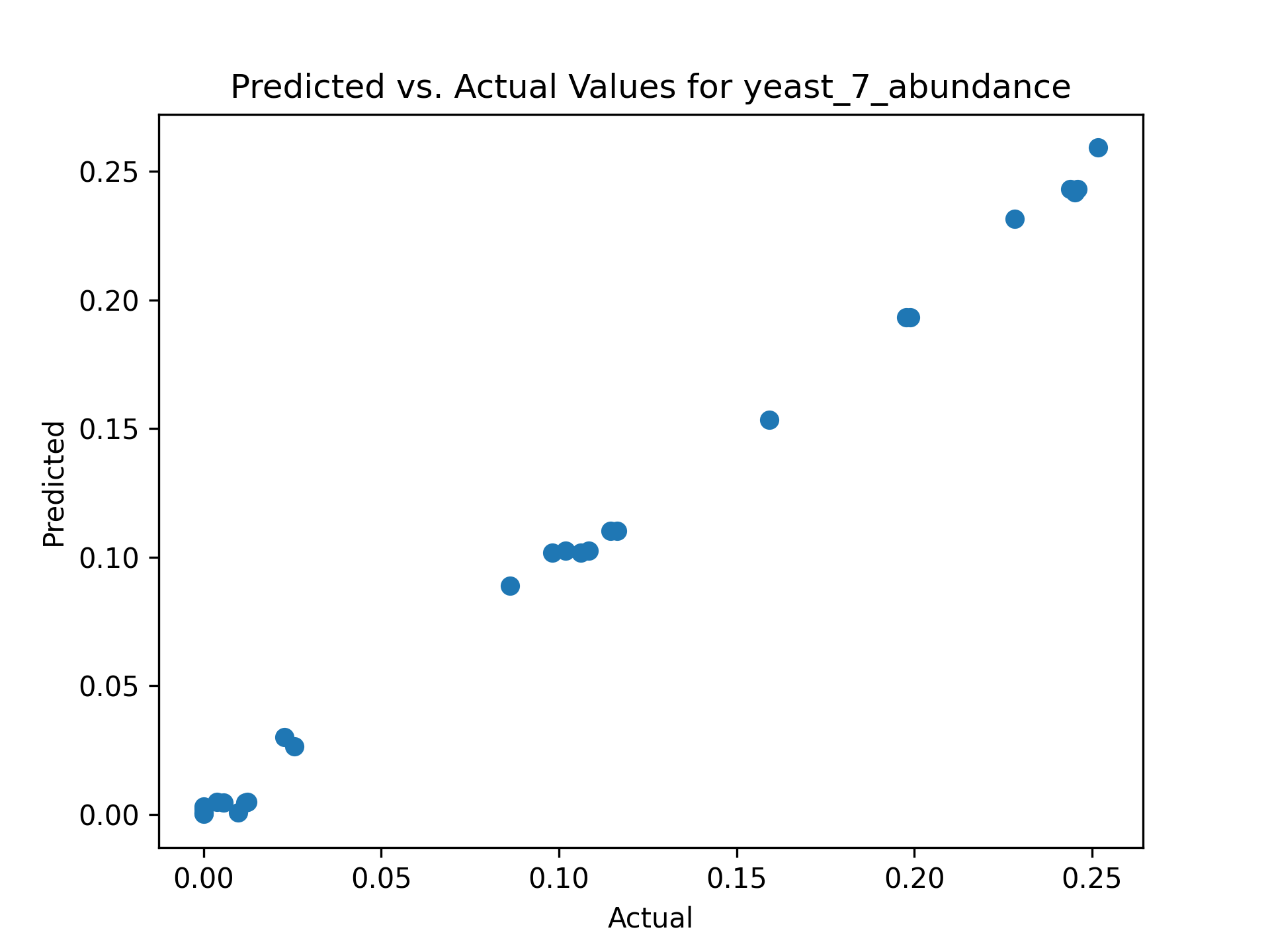

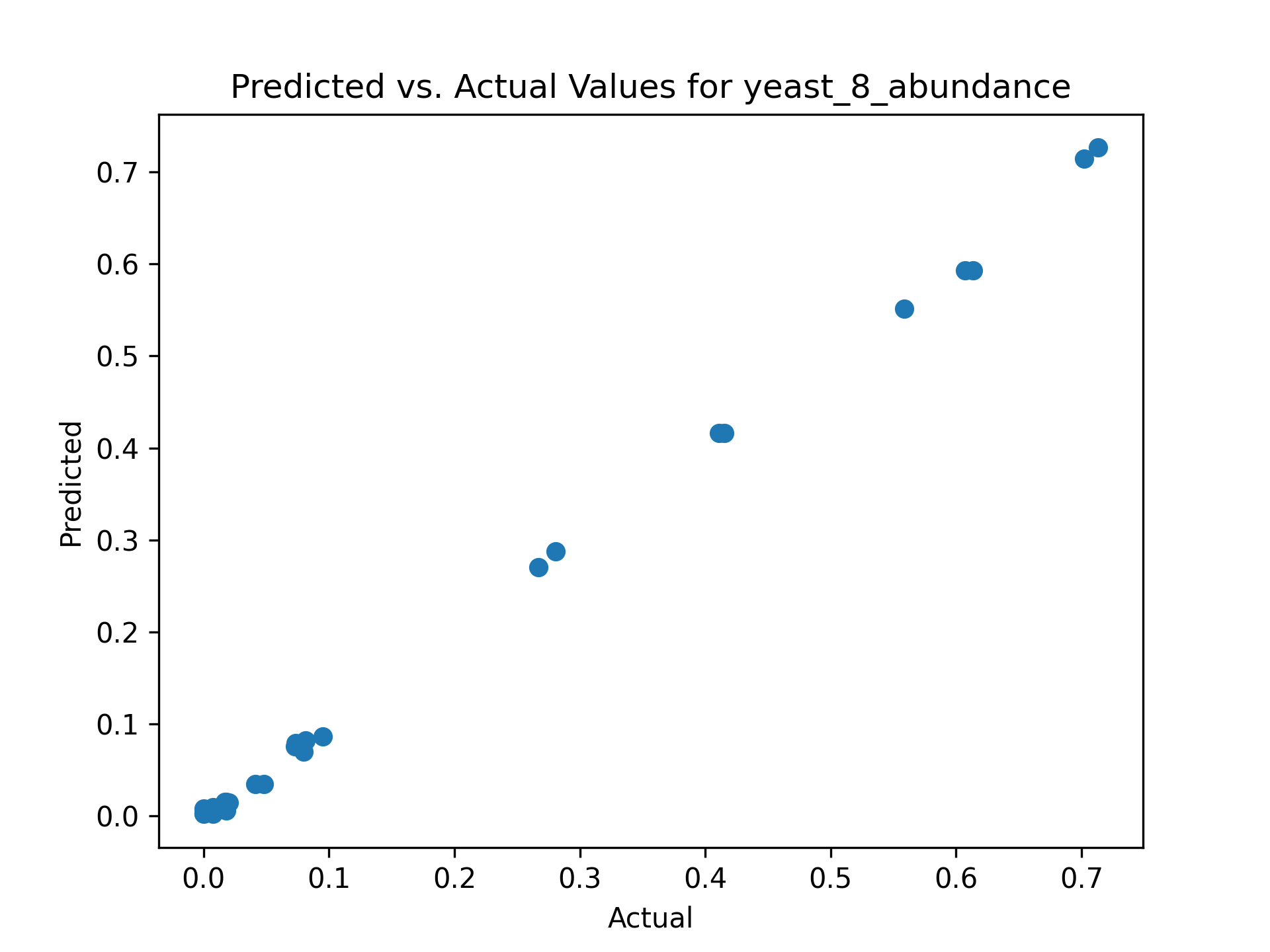

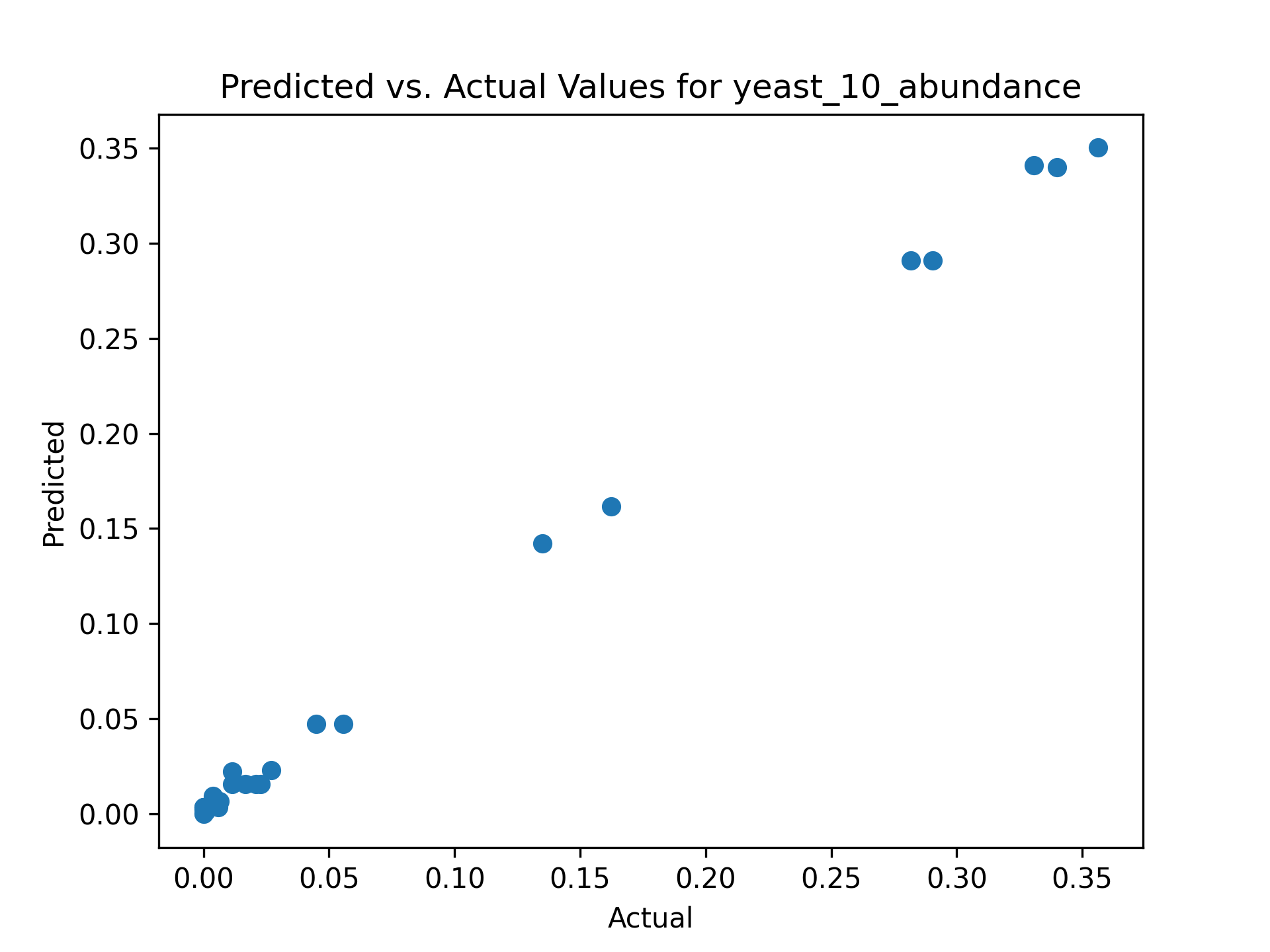


**A**

**B**

**C**

**D**

**E**

**F**

**G**

**H**

**I**

**Supp. Fig. 13.** Predicted versus actual value plots for each target variable in the Gradient Boosting Regressor model trained on the Bagheri et al dataset supplemented with synthesized data. Each subfigure (A-I) shows a scatter plot where each point represents a particular sample. The x-axis denotes the actual values, and the y-axis denotes the predicted values for each target. A perfect model would result in all points aligning along the diagonal line, which represents a one-to-one correspondence between predicted and actual values. Deviations from this line indicate prediction errors. Subfigures: (A) yeast_1_abundance, (B) yeast_2_abundance, (C) yeast_3_abundance, (D) yeast_4_abundance, (E) yeast_5_abundance, (F) yeast_6_abundance, (G) yeast_7_abundance, (H) yeast_8_abundance, (J) yeast_10_abundance.

Supplementary Code Files:

All code is available on Zenodo repository at the following DOI: 10.5281/zenodo.11384789.
